# Acute colitis during chronic experimental traumatic brain injury in mice induces dysautonomia and persistent extraintestinal, systemic, and CNS inflammation with exacerbated neurological deficits

**DOI:** 10.1186/s12974-020-02067-x

**Published:** 2021-01-18

**Authors:** Marie Hanscom, David J. Loane, Taryn Aubretch, Jenna Leser, Kara Molesworth, Nivedita Hedgekar, Rodney M. Ritzel, Gelareh Abulwerdi, Terez Shea-Donohue, Alan I. Faden

**Affiliations:** 1 Department of Anesthesiology and Shock, Trauma and Anesthesiology Research (STAR) Center, University of Maryland School of Medicine, 685 West Baltimore Street, MSTF #6-016, Baltimore, MD 21201 USA; 2 School of Biochemistry and Immunology, Trinity Biomedical Sciences Institute, Trinity College, Dublin, Ireland; 3 Division of Translational Radiation Sciences (DTRS), Department of Radiation Oncology, University of Maryland School of Medicine, Baltimore, MD USA

**Keywords:** TBI, DSS, Colitis, Neuroinflammation, Neurodegeneration, Neurobehavior, Systemic inflammation, Intestinal inflammation, Dysautonomia, Brain-gut

## Abstract

**Background:**

Disruptions of brain-gut axis have been implicated in the progression of a variety of gastrointestinal (GI) disorders and central nervous system (CNS) diseases and injuries, including traumatic brain injury (TBI). TBI is a chronic disease process characterized by persistent secondary injury processes which can be exacerbated by subsequent challenges. Enteric pathogen infection during chronic TBI worsened cortical lesion volume; however, the pathophysiological mechanisms underlying the damaging effects of enteric challenge during chronic TBI remain unknown. This preclinical study examined the effect of intestinal inflammation during chronic TBI on associated neurobehavioral and neuropathological outcomes, systemic inflammation, and dysautonomia.

**Methods:**

Dextran sodium sulfate (DSS) was administered to adult male C57BL/6NCrl mice 28 days following craniotomy (Sham) or TBI for 7 days to induce intestinal inflammation, followed by a return to normal drinking water for an additional 7 to 28 days for recovery; uninjured animals (Naïve) served as an additional control group. Behavioral testing was carried out prior to, during, and following DSS administration to assess changes in motor and cognitive function, social behavior, and mood. Electrocardiography was performed to examine autonomic balance. Brains were collected for histological and molecular analyses of injury lesion, neurodegeneration, and neuroinflammation. Blood, colons, spleens, mesenteric lymph nodes (mLNs), and thymus were collected for morphometric analyses and/or immune characterization by flow cytometry.

**Results:**

Intestinal inflammation 28 days after craniotomy or TBI persistently induced, or exacerbated, respectively, deficits in fine motor coordination, cognition, social behavior, and anxiety-like behavior. Behavioral changes were associated with an induction, or exacerbation, of hippocampal neuronal cell loss and microglial activation in Sham and TBI mice administered DSS, respectively. Acute DSS administration resulted in a sustained systemic immune response with increases in myeloid cells in blood and spleen, as well as myeloid cells and lymphocytes in mesenteric lymph nodes. Dysautonomia was also induced in Sham and TBI mice administered DSS, with increased sympathetic tone beginning during DSS administration and persisting through the first recovery week.

**Conclusion:**

Intestinal inflammation during chronic experimental TBI causes a sustained systemic immune response and altered autonomic balance that are associated with microglial activation, increased neurodegeneration, and persistent neurological deficits.

**Supplementary Information:**

The online version contains supplementary material available at 10.1186/s12974-020-02067-x.

## Background

Traumatic brain injury (TBI) is a chronic disease process that is characterized by primary and secondary injuries. Secondary injury begins within minutes of brain impact and can persist for months to years following the initial mechanical insult, resulting in progressive neurodegeneration, neuroinflammation, and neurobehavioral deficits [1–3]. TBI can also cause peripheral organ dysfunction, including gastrointestinal (GI) dysfunction. TBI-induced GI changes include mucosal injury, barrier disruption, and altered motility that underlie clinical symptoms of altered frequency of bowel movements, feeding intolerance, weight loss, and GI bleeding [4–6]. TBI patients who survived longer than 1-year post injury were 12 times more likely to have died of septicemia and 2.5 times more likely to have died of digestive conditions when compared to age-matched healthy cohorts of the general population [7]; yet little is known about the impact of GI disorders and diseases during the secondary injury phase of TBI.

The brain-gut axis is comprised of bidirectional communication pathways that contribute to physiological homeostasis, as well as the development and progression of pathological conditions affecting each organ system [8]. Brain-gut interactions are controlled largely by the autonomic nervous system (ANS), with the parasympathetic and sympathetic nervous systems providing reciprocal actions that serve to balance GI function. Brain-gut communication is also modulated by the immune system, with systemic immune responses playing an important role in posttraumatic brain pathophysiology [9, 10]. Patients with chronic neurodegenerative disorders, such as Parkinson’s disease, often report GI dysfunction, and emerging evidence indicates that GI disorders and diseases are associated with the development of neurological dysfunction [11–15]. Moreover, mood disorders and cognitive impairment occur in patients with inflammatory bowel disease (IBD) [16–18]. In preclinical models of IBD, mice exhibit increased anxiety-like behavior and deficits in declarative, working, and fear-motivated learning and memory [15, 19–23]. Induction of infectious colitis with the pathogenic bacteria, *Citrobacter rodentium*, at a chronic time point following experimental TBI exacerbated TBI-associated neuropathology and neuroinflammation [24]. However, the mechanisms by which enteric challenges or injuries contribute to secondary brain injury and long-term neurological outcomes following TBI remain unknown.

In the present study, we investigated the effect of chemically induced intestinal inflammation, initiated weeks after experimental TBI, on long-term changes in neuroinflammation, neuropathology, and neurobehavior. We used the well-characterized colitis model of dextran sodium sulfate (DSS), administered beginning 28 days after TBI in the drinking water over 7 days to induce acute colonic inflammation, followed by a return to normal drinking water for a recovery period to allow assessment of progressive changes in brain-gut interactions. Craniotomy alone, commonly used as a sham injury control in experimental TBI studies, is now recognized to cause mild brain injury with acute changes in inflammation, vasculature, and behavior [25–29]. Therefore, to address the potential confounding effect of brain injury in a sham experimental group, we included anesthetized, but unoperated, animals (Naïve) to serve as an additional control group. We performed behavioral testing to assess changes in fine motor coordination, declarative memory, spatial learning and memory, social behavior, and anxiety-like behavior. Neurodegeneration and microglial activation were measured by stereological assessment and assessed further at the molecular level using nanostring analysis. Our findings demonstrate that delayed intestinal inflammation during chronic TBI leads to persistent systemic inflammation and exacerbation of long-term pathological and behavioral outcomes.

## Methods

### Animals

Studies were performed using adult male C57BL/6NCrl mice (5 weeks old upon arrival; Charles River, Housing Area F50, Kingston, NY). As female mice are not as susceptible to the effects of DSS [30–32] and our previous study [24] examined male mice, this study was conducted with male mice only. Mice were housed in the animal care facility at the University of Maryland School of Medicine under a 12-h light-dark cycle, with ad libitum access to food and water. One week following arrival, all mice were subjected to a 3-week bedding mix protocol to minimize the influence of the microbiome using an established flora transfer regimen adapted from that described in the Jackson Laboratory DSS-protocol [33]. Briefly, a half cup of dirty bedding containing feces was removed from all cages and mixed in a sterile container. A half cup of the mixed dirty bedding was then redistributed into a clean cage, and the mice were placed into these cages. This process was carried out once a week on cage change day. Although our experimental design attempted to minimize effects of the microbiome, which has been shown to affect CNS injury outcomes [34, 35], we did not assess how microbiome changes due to TBI or DSS might affect outcomes. All behavioral and surgical procedures were carried out in accordance with protocols approved by the Institutional Animal Care and Use Committee at the University of Maryland School of Medicine.

### Experimental design

**Fig. 1:**
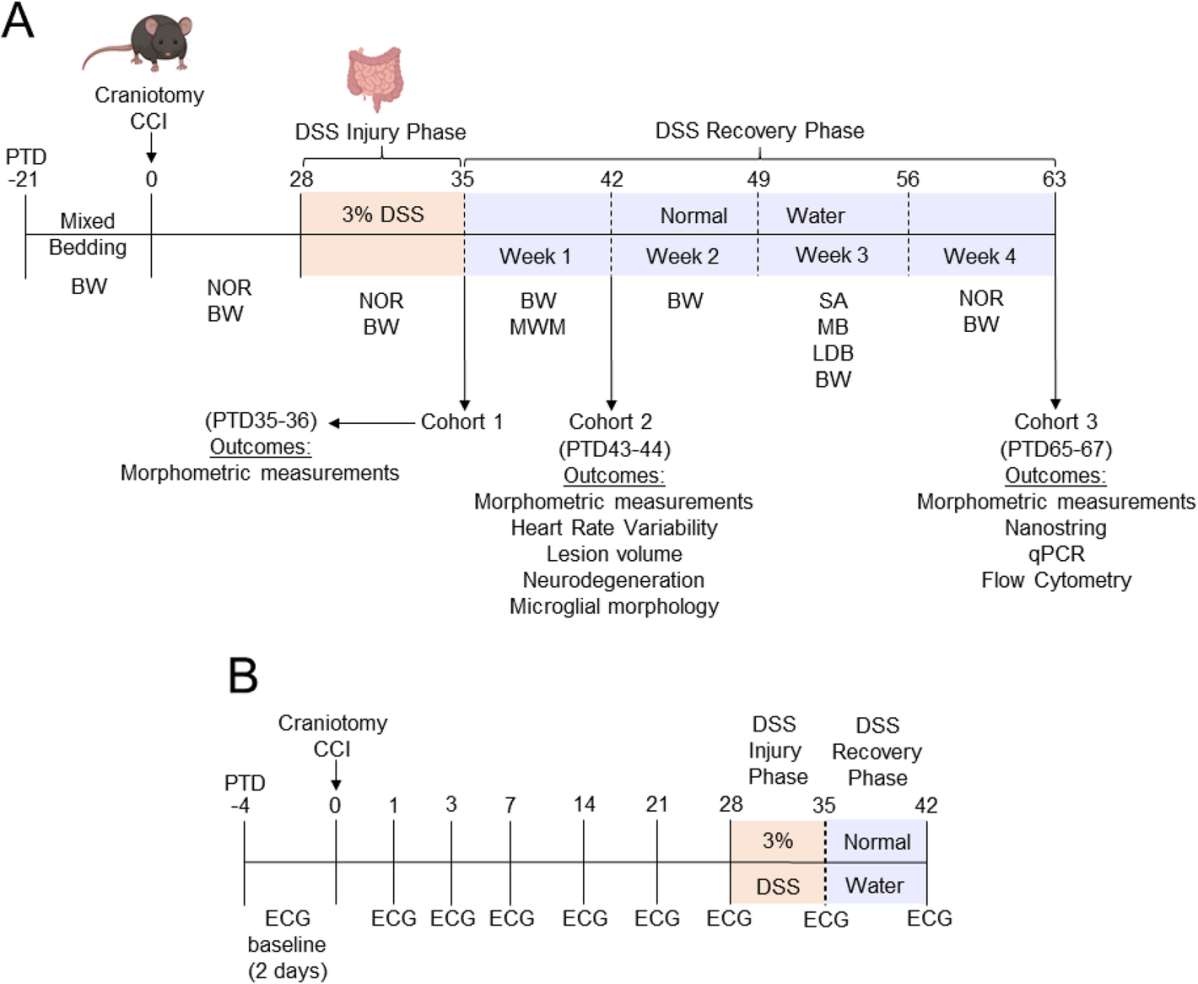
Experimental timelines. Experimental timeline for *cohorts* 1, 2, and 3 of mice **a**. Adult male C57BL/6Nrcl mice arrived at 5 weeks of age. After 1 week of acclimation to the animal facility all mice underwent a 3-week bedding mix protocol following which, at 9 weeks of age, mice underwent either anesthetic exposure only (Naïve), craniotomy (Sham), or craniotomy+controlled cortical impact, CCI (TBI). At 4 weeks following surgery, mice either continued normal drinking water or were administered 3% DSS in drinking water for 7 days to induce intestinal inflammation, followed by a return to normal drinking water for an additional 7 (*cohort* 2) or 28 (*cohort* 3) days to recover. Neurobehavioral testing was conducted throughout the study to assess changes in motor function (BW), cognition (MWM, NOR), social behavior (SA), and anxiety-like behavior (MB, LDB). *Cohorts* 1 and 2 were sacrificed at PTD 35-36 and 42-43, respectively. *Cohort* 3 underwent all behavioral testing and were sacrificed on PTD 64-68. Colons were collected for morphometric analyses. Blood, spleens, and mesenteric lymph nodes were collected for flow cytometry. Brains from *cohorts* 1 and 2 were collected for histological analyses. Brains from *cohort* 3 were dissected into specific subregions for molecular analyses. Additionally, *cohort* 2 mice underwent ECG for heart rate variability analysis at various timepoints throughout the study to assess changes in autonomic balance **b**. Abbreviations: BW, beam walk; CCI, controlled cortical impact; DSS, dextran sodium sulfate; ECG, electrocardiography; LDB, light-dark box; MB marble burying; MWM, Morris water maze; NOR, novel object recognition; PTD, post-TBI day; qPCR, quantitative polymerase chain reaction; SA, Crawley’s three-chamber social approach task. Mouse and intestine graphics created with Biorender.com

At 9 weeks of age, male C57BL/6NCrl mice (*n* = 8–12/group *cohort 1*; *n* = 14–21/group *cohort 2*; *n* = 15–21/group *cohort 3*) were subjected to either isoflurane exposure (Naïve), craniotomy (Sham), or controlled cortical impact (CCI). On post-TBI day (PTD) 28, animals were randomly placed into two groups; one group not subjected to intestinal inflammation (normal drinking water) and a second group subject to intestinal inflammation (3% dextran sodium sulfate via drinking water). Dextran sodium sulfate (DSS) was administered daily for 7 days, followed by a return to normal drinking water for an additional 7 to 28 days. Neurobehavioral testing was performed prior to, during, and following DSS administration to assess motor and cognitive function, social behavior, and anxiety-like behavior in *cohort 3* (Fig. 1a). Additionally, *cohort 2* mice underwent electrocardiography (ECG) to collect heart rate variability (HRV) data for analysis of autonomic balance (Fig. 1b). Following completion of the study on PTD 35-36 (*cohort 1*), PTD 42-43 (*cohort 2*), or PTD 65-67 (*cohort 3*), mice were weighted, anesthetized with 4.5% isoflurane and 1 ml of blood was collected via the retro-orbital plexus using heparin-coated micro-hematocrit capillary tubes (22-362-566, Thermo Scientific, Pittsburg, PA) for use in flow cytometry. Isoflurane was then reduced to 1.5% and the mesenteric lymph nodes (mLNs), and spleen were removed and weighted. Additionally, the number of nodes comprising the mLNs collected for each mouse was recorded. The colon was removed, morphometric analyses taken (length, weight), and the mice were then either transcardially perfused with 100 ml ice-cold 0.9% saline (*cohort 3*) or perfused with 100 ml ice-cold 0.9% saline, followed by 100 ml of 4% paraformaldehyde (*cohorts 1 and 2*). Fixed brains were removed and post-fixed in 4% paraformaldehyde for 24 h, followed by cryoprotection in 20% sucrose for 48 h and finally stored in 30% sucrose until processed for histological analyses (*cohorts 1 and 2*). Brains from mice perfused with saline alone (*cohort 3*) were removed and rapidly dissected on a cutting mat chilled to 4 °C. The contralateral and ipsilateral hippocampus was flash frozen in liquid nitrogen and stored at − 80 °C for later use in RNA/Nanostring analysis. Spleens, mLNs, colons, and blood from a subset of mice from *cohort 3* were taken and processed for flow cytometry. Body weights from all mice were recorded weekly upon arrival and then daily beginning the day of surgery through the end of the study at the same time every day. The percent body weight lost and regained during and following DSS administration, through the end of the study, was calculated using the following formula: *(body weight (g) / PTD28 body weight (g)) × 100*. Body weights for all mice on the first day of DSS administration (PTD28) were obtained prior to adding DSS into the drinking water.

### Controlled cortical impact (CCI)

CCI was delivered using a custom-designed CCI injury device fitted with a microprocessor-controlled pneumatic impactor tip with a diameter of 3.5 mm [2, 36, 37]. Briefly, mice were anesthetized with isoflurane evaporated in a gas mixture containing 70% nitrous oxide (N_2_O) and 30% O_2_ administered through a nose mask (induction at 3%, maintenance at 1.5%). A 10-mm midline incision was made over the skull and the skin and fascia were reflected. A 4-mm craniotomy was made on the central aspect of the left parietal bone. The impactor tip was extended to its full stroke distance (44 mm), positioned over the surface of the exposed cortex, and set to impact the cortical surface. Moderate-to-severe level CCI was then induced by using an impactor velocity of 6 meters/second (m/s) and deformation depth of 2 mm. The incision was closed with 6–0 silk sutures, anesthesia terminated, and the mouse was placed into a heated chamber for 45 min postinjury to maintain core body temperature. Sham mice underwent the same procedure as CCI mice without receiving the impact. Naïve mice received isoflurane exposure only. Timing of isoflurane exposure for naïve mice was based on exposure times from craniotomy and CCI surgeries.

### Dextran sodium sulfate (DSS) administration

DSS was purchased from MP Biomedical (product # 0216011025, Solon, OH). Administration occurred via drinking water in feeding bottles fitted with metal drinking tubes (TD-100 and 5.5R, Ancare, Bellmore, NY) for 7 days (DSS injury phase) at a concentration of 3% (w/v). After 7 days, mice were returned to normal drinking water for an additional 7 (*cohort 2*) or 28 (*cohort 3*) days (DSS recovery phase) (Fig. 1a). Daily measuring of liquid intake was performed to monitor consistency of DSS consumption and exposure between DSS-treated mice. The daily amount of liquid consumed by each cage of mice was divided by the number of mice in each cage to approximate the amount of liquid consumed per mouse.

### Disease activity index (DAI) score

The severity of DSS injury was measured by a composite score of three criteria: (1) percent body weight lost, (2) presence of blood in stool, and (3) stool consistency. Each animal was assigned a score of 1–4 for each criterion and the scores were added together for the final DAI score as previously described [38]. Presence of fecal blood was assessed using the Hemoccult II® SENSA® Fecal Occult Blood Test Systems (10012-016, WVR, Radnor, PA). DAI scores for all mice were carried out at the end of the DSS injury phase (PTD35) and each recovery week (PTD 42, 49, 56, 63).

### Electrocardiography (ECG)

ECG was performed on 2 days prior to brain injury (baseline day, BLD, 1 and 2). After brain injury (PTD0), ECG was performed on PTD1, 3, 7, 14, 28, 35, and 42. Briefly, mice were anesthetized using isoflurane evaporated in 2–2.5% oxygen (O_2_, flow rate 0.81/min), administered through a nose mask (induction at 3%, maintenance at 1.5%) and positioned supine with paws taped to the electrode pad coated with lubricant. Three 3-min recordings were taken using the Vevo 1100® ultrasound machine (Visual Sonic, Toronto, Canada). ECG data was then converted to an excel format and input into the Kubios HRV software (version 3.4, Kubios Oy.). Low-frequency (LF) and high-frequency (HF) values obtained from the data output were then used to calculate the LF/HF ratios from each recording and the ratios from the three recordings averaged [39].

### Neurobehavioral testing

All neurobehavioral testing was conducted at night under red light during the animals’ wake cycle, with testing beginning 1 h after lights out after habituating to the testing room. Computer screens were covered with red, transparent plastic film (4331997009, Amazon, Seattle, Washington). Order of mice on any given behavioral testing day was pseudo-randomly determined using a random list generator (random.org). Temperature, sound, and light levels were continually monitored using a thermometer, decibel meter (Extech 407730 Digital Sound Level Meter, Amazon, Seattle, Washington), and luminometer (Extech LT300 Light Meter, Amazon, Seattle, Washington) and typically averaged 23-24°C, 45 dB, and 35 lux unless otherwise stated.

### Motor function

Fine motor coordination was assessed using the beam walk test, as previously described [36]. Briefly, mice were placed at the end of a wooden beam (5 mm wide, 120 mm long), and the number of slips (foot faults) of the right hind limb was recorded over 50 steps. Mice were trained on the beam walk for 4 days prior to surgery and tested again on days 1, 3, 7, 14, 21, 28, 32, 35, 39, 42, 56, and 63 post-TBI. Mice that did not meet standard criterion for beam walk, ≤ 10-foot faults, prior to surgery were excluded from the final data analysis (TBI: *n* = 1). Gross motor function was also assessed during the habituation stage of novel object recognition testing (distance travel, average speed) and during Morris water maze testing (swim speed) using Any-Maze software (Stoelting Company, Wood Dale, IL).

### Novel object recognition (NOR) task

Declarative memory in mice was assessed by performing NOR testing [40, 41]. *Cohort 3* mice underwent repeated NOR testing at three points throughout the study on PTD 24-27 (pre-DSS), PTD 31-33 (DSS injury phase), and PTD 59-61 (DSS recovery phase). Mice underwent two habituation days consisting of one 10-min trial/day to acclimate to the testing arena (22.5 × 22.5 cm, black plexiglass walls). Twenty-four hours following the second habituation day, mice underwent object familiarization in which the mice were placed into the testing arena with two similar objects positioned equidistant apart from each other and the arena walls and allowed to explore freely for 5 min. Twenty-four hours later, mice underwent novel object (NO) testing in which one familiar object was replaced with a novel object and allowed to freely explore for 5 min. Objects utilized in this study consist of star Rubik cubes (47010, Toys’R’Us); wooden hearts, stars, diamond, and flowers (5F63E8C, Toys’R’Us); mini blue ping-pong balls (FON-10187-S100, Amazon, Seattle, Washington); blue heart Rubik center (B01GGG1XI4, Amazon, Seattle, Washington); and plastic interlocking spheres and circles (B06XH69FS2, Amazon, Seattle, Washington). A new object set was used during each NOR test such that objects presented during pre-DSS, DSS injury phase, and DSS recovery phase were completely different from each other (e.g., pre-DSS: plastic interlocking spheres and circles; DSS injury phase: wooden heart/star and diamond/flower, DSS recovery phase: blue heart Rubik center and blue ping-pong balls). Novel/familiar objects and side in which the novel object was positioned were balanced across all experimental groups at each testing time point to control for any potential object and side biases. Testing was recorded using Any-Maze software (Stoelting Company, Wood Dale, IL). Object interaction included time spent sniffing or placing front paws on, while facing, the object. Time spent chewing and climbing and sitting on top of objects was not considered exploration and excluded from final scores. Preference index (PI) for the novel versus familiar object was calculated using the following equation: *(Time Spent Exploring NO/Time Spent Exploring Both Objects) × 100*.

### Morris water maze (MWM)

Hippocampal-dependent spatial learning and memory was assessed using the MWM [42]. Testing included three stages: (1) *learning acquisition* consisting of hidden platform training, (2) reference memory consisting of *probe trial* with the platform removed, and (3) *visual acuity* consisting of a 60-s trial with a flagged platform. Briefly, a white circular tank (100 cm in diameter) was filled with water (23 ± 2 °C) and various extra-maze cues (blue and yellow square, circle, diamond, star) were positioned on the walls surrounding the testing area. A transparent plexiglass platform (10 cm in diameter) was submerged to a depth of 0.5 cm below the surface of the water. Acquisition stage testing was carried out over four consecutive days (PTD 38-41) during which mice were trained to find the hidden submerged platform located in the northwest (NW) quadrant of the tank. Mice performed four 90-s trials per day, with an inter-trial interval of 30 min. Entry points (northeast, east, south, southwest) and retrieval points (north, south, east, west) were randomly assigned for each trial each day with mice being lowered gently into the tank, facing the tank wall. Mice were allowed 90 s to find the platform with an escape being considered successful if the mouse located and remained on the platform for 5 s. Mice unable to locate the platform within the allotted time were guided to the platform and allowed to remain on the platform for 15 s prior to removal from the tank. After removal from the tank, mice were gently dried with plush towels (18006-1712, Dollar Tree, Chesapeake, VA) and placed into open cages lined with towels, positioned partially under a heat lamp. Once dry, mice were returned to their home cages until the next trial. Twenty-four hours following the final acquisition day (PTD 42), reference memory was tested by carrying out a 60-s probe trial in which the submerged platform was removed from the tank. Mice were released into the tank in the southeast quadrant and removed promptly after completion of the trial. Approximately 1 h after completion of the probe trial, mice underwent the final stage with a 60-s visual acuity trial to ensure mice did not have visual impairments. A flagged platform was positioned in the southeast quadrant and mice were released into the tank in the northwest quadrant. All trials were recorded using Any-Maze software (Stoelting Company, Wood Dale, IL). Latency to escape (acquisition trials, visual acuity trial) and time spent in the target quadrant (probe trial) were recorded. Analysis of search strategy utilized by mice during the probe trial was performed and classified into three categories: (a) spatial, (b) non-spatial systematic, and [3] repetitive looping [43]. Spatial search strategy was defined as swimming directly to the platform or to the target quadrant and searching for the platform. Non-spatial systematic search strategy was defined as searching the interior or entirety of the tank without spatial bias prior to finding the platform. Repetitive looping search strategy was defined as repetitive circular swimming around the tank. Mice that learned to float were excluded from the final results (Naïve:1, Sham:1, Naïve+DSS:1).

### Three-Chamber Social Approach (SA) Task

Also known as Crawley’s sociability and social novelty test, this task is based on rodents’ innate tendency to investigate a novel congener over a familiar one [44, 45]. The test arena consisted of a rectangular box made from clear plexiglass, divided into three equal-sized chambers (19 × 45 × 35cm). Access to the two side chambers from the center chamber is allowed by 7 × 7cm openings. Two metal wire pencil cups were used to hold the stimulus mice (TXBT, Amazon, Seattle, Washington). This test is composed of three sequential 10-min stages: stage 1: *habituation* in which the mice are acclimated to the testing chamber, stage 2: *sociability* in which the mice are exposed to stimulus mouse 1, and stage 3: *social novelty* in which mice are exposed to stimulus mouse 1 and novel stimulus mouse 2. Briefly, test mice were first placed into the center chamber with the empty wire cups in both side chambers and allowed to freely explore all chambers for 10 min. After 10 min, the test mice were guided back into the center chamber and access to side chambers blocked. A stimulus mouse (stimulus mouse 1) was placed into one of the wire cups while a black wooden circle was placed into the remaining wire cup. Access to the side chambers was granted and the test mice were allowed to explore freely for 10 min. After 10 min, the test mice were again guided back into the center chamber and access to side chambers blocked. A second stimulus mouse (stimulus mouse 2) was placed into the wire cup previously holding the object, access to the side chambers granted and test mice were allowed to explore freely for 10 min. After 10 min, the stimulus and test mice were returned to their home cages. The testing arena and wire cages were wiped clean in between each test run. All tests were recorded using a video recorder (1960C002, Amazon, Seattle, Washington). Time spent exploring the empty cups, object, and stimulus mice in each stage were measured, and the preference index for the stimulus mouse (mouse 1) versus the object (stage 2) and novel stimulus mouse (mouse 2, stage 3) versus the familiar stimulus mouse (stimulus mouse 1) was calculated using the following equations: *[(time spent interacting with stimulus mouse 1 / (time spent interacting with stimulus mouse 1 + time spent exploring the object))]* × *100* ; *[(time spent interacting with stimulus mouse 2 / (time spent interacting with stimulus mouse 1 + time spent exploring stimulus mouse 2)]* × *100.* Stimulus mice used were all male C57Bl/6J mice and of a similar age/weight to the experimental mice. These mice were obtained from our breeding colony and had not undergone any behavioral testing or surgical procedures prior to running this behavior task. Three days prior to beginning this task, all stimulus mice were habituated to the wire cups for one 10-min trial on the first day, one 20-min trial on the second day, and one 10-min and 20-min trial on the third day. Stimulus mice used, and chamber in which the stimulus mice and object were placed, were balanced across all stages and experimental groups. Mice underwent testing during the third week of the DSS recovery phase (PTD 50-54). Mice that did not interact with the stimulus mice/object exhibited chamber biases or learned to jump out of the testing arena were excluded from final results (TBI: *n* = 2, Naïve+DSS: *n* = 1; Sham+DSS: *n* = 3, TBI + DSS: *n* = 1).

### Marble burying (MB)

Repetitive and anxiety-like behavior was assessed by performing marble burying [46, 47]. A sterile cage with an open top lid (26 × 37 × 13 cm, Ugo Basile, Gemonio, Italy) was filled with sterile bedding to a depth of 5 cm and twenty black glass marbles (754316778107, Amazon, Seattle, Washington) were positioned equidistant apart on top of the bedding in a 4 × 5 arrangement. Mice were placed into the cage and allowed to freely explore for 30 min after which they were returned to their home cages. The number of marbles buried by at least two thirds was recorded. Tests were recorded using Any-Maze software. A separate test cage of bedding was prepared for each cage of mice, with the bedding being mixed after completion of every test and replaced completely after every third mouse. Marbles were soaked in 70% ethanol following every test and dried completely prior to being used again. Mice that remained immobile during the test or learned to jump out of the arena were excluded (Naïve: *n* = 1, TBI: *n* = 1).

### Light-Dark Box (LDB)

LDB testing utilizes rodents’ innate aversion to brightly light open spaces and desire to explore novel environments to assess anxiety-like behavior [48]. The testing chamber is composed of two equally sized chambers (40 × 20 × 35 cm) connected by a small 7 cm opening through which the mice can pass freely. The light chamber was constructed from clear plexiglass and illuminated brightly with two white lights (350 lux) positioned 10 cm above the open chamber, while the dark chamber was constructed from black plexiglass and enclosed with a removable black plexiglass top. Mice were placed into the light chamber, facing away from the opening to the dark chamber, and allowed to freely explore both chambers for 10 min. After 10 min, the mice were returned to their home cage and the testing arena wiped clean. All tests were recorded using Any-Maze software, and mice were scored according to the time spent in each chamber, latency to enter light chamber from dark chamber. Animals were tested once during the third week of the DSS recovery phase (PTD 55-56).

### Lesion volume

Assessment of lesion volume was performed on 60 μm fixed coronal brain sections stained with cresyl violet (FD NeuroTechnologies, Baltimore, MD; *n* = 8–10/group). The Cavalieri method was used to quantify lesion volume using StereoInvestigator software (MBF Biosciences, Williston, VT), as previously described [43]. Briefly, the lesion volume was quantified by outlining the missing tissue on the injured hemisphere using the Cavalieri estimator with a grid spacing of 0.1 mm. Every fourth section from a total of 72 sections was analyzed beginning from a random start point.

### Neuronal loss

Analysis of hippocampal neuronal cell loss was performed on 60 μm fixed coronal brain sections stained with cresyl violet, with every fourth 60 μm section between − 1.34 and − 2.54 mm and − 2.7 to − 3.16 mm from bregma, beginning at a random starting point, being analyzed (*cohort 2*, *n* = 8–10/group). A total of 6 sections per brain were analyzed. Neuronal cell loss was quantified using a Leica DM4000B microscope (Leica Microsystems, Exton, PA) with the StereoInvestigator software (MBF Biosciences, Williston, VT) by counting the number of surviving neurons using the optical fractionator method of unbiased stereology. The volume of the regions counted was determined using the Cavalieri method. Results are expressed in terms of cellular density (cells per mm^3^) [36, 49]. Neuronal cell counts were not performed in the CA1 or CA2/3 subregions in TBI mice as portions of either or both were missing due to the injury.

### Microglia morphological analysis

Morphological activation states of microglia in the hippocampus and cortex were assessed by performing ionized calcium-binding adapter molecule 1 (Iba-1) staining on 60 μm fixed coronal sections from *cohort 2* mice (*n* = 8–10/experimental group). Briefly, free-floating sections were permeabilized with 0.3% triton X-100 in 1× phosphate-buffered saline (PBS) for 10 min, washed in 1× PBS, endogenous peroxidase activity block by incubating with 3% hydrogen peroxide/80% methanol for 10 min, washed in 1× PBS, blocked in blocking buffer (3% bovine serum albumin (BSA)/10% goat serum/0.1% Triton-X100/1× PBS) for 1 h, incubated overnight rocking at 4 °C with rabbit anti-Iba-1 (1:3000; 019-19741, Wako Chemicals, Richmond, VA) in blocking buffer, washed in 1× PBS, and incubated with biotinylated goat anti-rabbit IgG antibody (1:2000, BA-1000, Vector Laboratories, Burlingame, CA) in blocking buffer for 2 h at room temperature. Sections were then incubated in an avidin-biotin-horseradish peroxidase solution (Vectastain Elite ABC kit, PK-6100, Vector Laboratories) for 1 h and then incubated with 3,3-diaminobenzidine/Nickel (II) Chloride (D5905-50TAB and 339350-50G, Sigma-Aldrich, St. Louis, MO) for color development. Sections were then washed in 1× PBS, mounted, dried, and counterstained with cresyl violet. MB Biosciences StereoInvestigator software was used to count and classify the number of ipsilateral hippocampal microglia based on morphologic phenotypes (ramified and activated: hypertrophic, bushy) using the optical fractionator method as previously described [43].

### Nanostring analysis

RNA was extracted and purified from frozen ipsilateral hippocampal tissue using the miRNeasy Mini Kit according to the manufacturer’s instructions (QGN-217004, Qiagen, Hilden, Germany). Total RNA was diluted to 20 ng/μl and probed using an nCounter© Mouse Neuropathology panel (Nanostring Technologies, Seattle, WA) profiling 770 genes across six fundamental themes of neurodegeneration: neurotransmission, neuron-glia interaction, neuroplasticity, cell structure integrity, neuroinflammation, and metabolism. Pairwise differential expression and pathway analyses were performed using NanoStringDiff (v3.6.0) [50] with raw cell counts including negative and positive controls and housekeeping genes from NanoString nCounter. As the analysis was comprised on only two groups, only one comparison was made: Sham vs. Sham+DSS. Differentially expressed genes were defined as those having a *p* value < 0.05 compared to Sham. Volcano of expression data, histograms of pathway scores, and violin plots of normalized gene counts with median and upper/lower quartiles annotated were generated using Prism v8.4.2 (GraphPad, San Diego, CA).

### Quantitative PCR analysis

RNA extracted and purified from frozen ipsilateral hippocampal tissue for nanostring analysis was used for qPCR analysis. cDNA was transcribed from 1 μg RNA using the Verso cDNA kit following the manufacturer’s instructions with AB1453B (Thermo Scientific, Pittsburg, PA) and the T100 Thermo Cycler (Bio-Rad, Hercules, CA). Quantitative analysis of gene expression was performed using Taqman technology, as previously described [51]. Target mRNAs included TaqMan gene expression assays for *Mmp2* (matrix metalloprotease 2) Mm00439498_m1; *Arc* (activity-regulated cytoskeleton-associated protein) Mm00479619_g1; *Psmb9* (proteasome 20S subunit beta 9) Mm00479004; *Ptgs2* (prostaglandin synthase 2) Mm00478374; *Tspo* (translocator protein) Mm00437828_m1; *Gfap* (glial factor activating protein) Mm01253033_m1; (cluster of differentiation 68) Mm00839636_g1; *Cybb* (cytochrome b-245 beta chain, NOX2) Mm01287743_m1; *Il1rn* (Interleukin 1 receptor antagonist) Mm00446186_m1; *Gapdh* (glyceraldehyde-3-phosphate dehydrogenase) Mm99999915_g1 (Thermo Fisher Scientific, Pittsburg, PA). Analysis was performed using the ABI 7900 HT FAST Real Time PCR machine (Applied Biosystems, Carlsbad, CA) with the following parameters: 50 °C for 2 min; 95 °C for 10 s, and 60 °C for 1 min; 40 cycles. Gene expression was normalized to GAPDH and compared to the control sample to determine relative expression values by 2^−ΔΔ*Ct*^ method.

### Flow cytometry

A 1-cm segment of the proximal colon was flushed with ice-cold 1X PBS to remove fecal debris, weighted, cut into approximately 5-mm-thick strips, and placed into a 5 mL digestion solution containing 1.5 mg/ml collagenase A (Roche, 10103586001) and 0.4 mg/ml DNase 1 (Roche, 4716728001) in Hank’s Buffered Saline Solution (HBSS) with 5% fetal bovine serum (FBS) and 10 mM 2-[4-(2-hydroxyethyl)piperazin-1-yl] ethanesulfonic acid (HEPES). Samples were then incubated at 37 °C with continuous shaking at 250 rpm for 1 h with gentle vortexing every 15–20 min. Upon complete digestion, 25 mL of 1× PBS was added and the samples were vortexed at maximal speed for 30 s. The resulting cell suspension was filtered through a 70-μM cell strainer, pelleted, washed in 1× PBS with 5% fetal bovine serum (FBS), blocked, and stained as indicated below [52]. After sample collection, cell count data were normalized to tissue weight (weight range = 40.5–120.1 mg; average weight = 65.4 mg). The spleen and mesenteric lymph nodes were processed by mechanical disruption on a 70-μm filter screen using with RPMI (Lonza Group, Basel, Switzerland) medium. Red blood cell lysis was achieved by successive 10-min incubations with Tris-ammonium chloride (Stem Cell Technologies, Vancouver, Canada). All blood, colon, and mLNs cells were then transferred into FACS tubes and placed on ice until blocking. Splenocytes were subsequently washed and resuspended in a total of 5 ml of RPMI from which 500 μl was then transferred into FACS tubes. Leukocytes were washed and blocked with mouse Fc Block (clone 93, eBioscience, San Diego, CA) prior to staining with primary antibody-conjugated fluorophores (CD45-Bv421 (30-F11), CD11b-APC-Cy7 (M1/70), Ly6C-APC (HK1.4), CD115-AF488 (AFS98), Ly6G-PE (1A8), MHCII- PerCP-eF710 (M5/114.15.2), CD11c-PE-Cy7 (N418), CD3e-PE-Cy7 (145-2C11), CD4-FITC (GK1.5), and CD8a-AF700 (53-6.7) which were purchased from Biolegend (San Diego, CA, USA). For live/dead cell discrimination, a fixable viability dye, Zombie Aqua^TM^ (Biolegend), was dissolved in DMSO according to the manufacturer’s instructions and added to cells in a final concentration of 1:200. Data were acquired on a LSRII using FACsDiva 6.0 (BD Biosciences, San Jose, CA, USA) and analyzed using FlowJo (Tree Star, San Carlos, CA, USA). A standardized gating strategy was used to identify leukocyte subsets as previously described [10]. The total number of cells per mLN sample was divided by the number of mLNs (1–3) collected for that sample. Cell count estimations were performed using CountBright^TM^ absolute counting beads (Invitrogen, Carlsbad, CA) according to the manufacturer’s instructions. Final data were presented as cells/μl.

### Statistical analysis

Blinding within the study was performed as follows: (1) individual who administered DSS was blinded to injury group, (2) behavioral and stereological analyses were performed by individuals blinded to injury and treatment groups, and (3) DAI scores and morphometric analyses were performed by individuals blinded to injury and treatment groups. Quantitative data were expressed as mean ± standard error of the mean (s.e.m.). Liquid consumption, DAI scores, weight loss, beam walk, MWM acquisition, and heart rate variability (LF/HF) were analyzed by repeated measures two-way analysis of variance (ANOVA) to determine the interactions of time and injury and DSS administration, followed by post hoc adjustments using Tukey’s multiple comparison test. Pre-DSS distance traveled, speed, object, and novel object preference (NOR) were analyzed by one-way ANOVA, followed by post hoc adjustments using Tukey’s multiple comparison test. Distance traveled (NOR), mean speed (NOR), object and novel object preference (NOR), time spent in the target quadrant (MWM probe trial), swim speeds (MWM probe trial), latency to escapes during visual acuity (MWM), stimulus mouse preference (SA), exploration times (NOR, SA), number of marbles buried (MB), time in the light chamber (LDB), entries into the light chamber (LDB), latency to re-enter the light chamber from the dark chamber (LDB), and all morphological analyses were analyzed by two-way ANOVA, followed by post hoc adjustments using Tukey’s multiple comparison test. The MWM search strategy was analyzed using a chi-square analysis. Neuronal cell counts and microglia morphological/cell counts were analyzed by two-way ANOVA, followed by Tukey’s post hoc. Stereological data examining lesion volume and nanostring pathway scores and gene expression data were analyzed using an unpaired, two-tailed Student *t* test. Data that did not pass normality were subjected to either a square root, cube root, or Box-Cox transformation for statistical analysis. Statistical analyses were performed using GraphPad Prism program, Version 8.4.2 for Windows (GraphPad Software, San Diego, CA, USA) with a *p* < 0.05 being considered statistically significant.

## Results

### Prior chronic TBI does not affect DSS disease severity or delay recovery

**Fig. 2:**
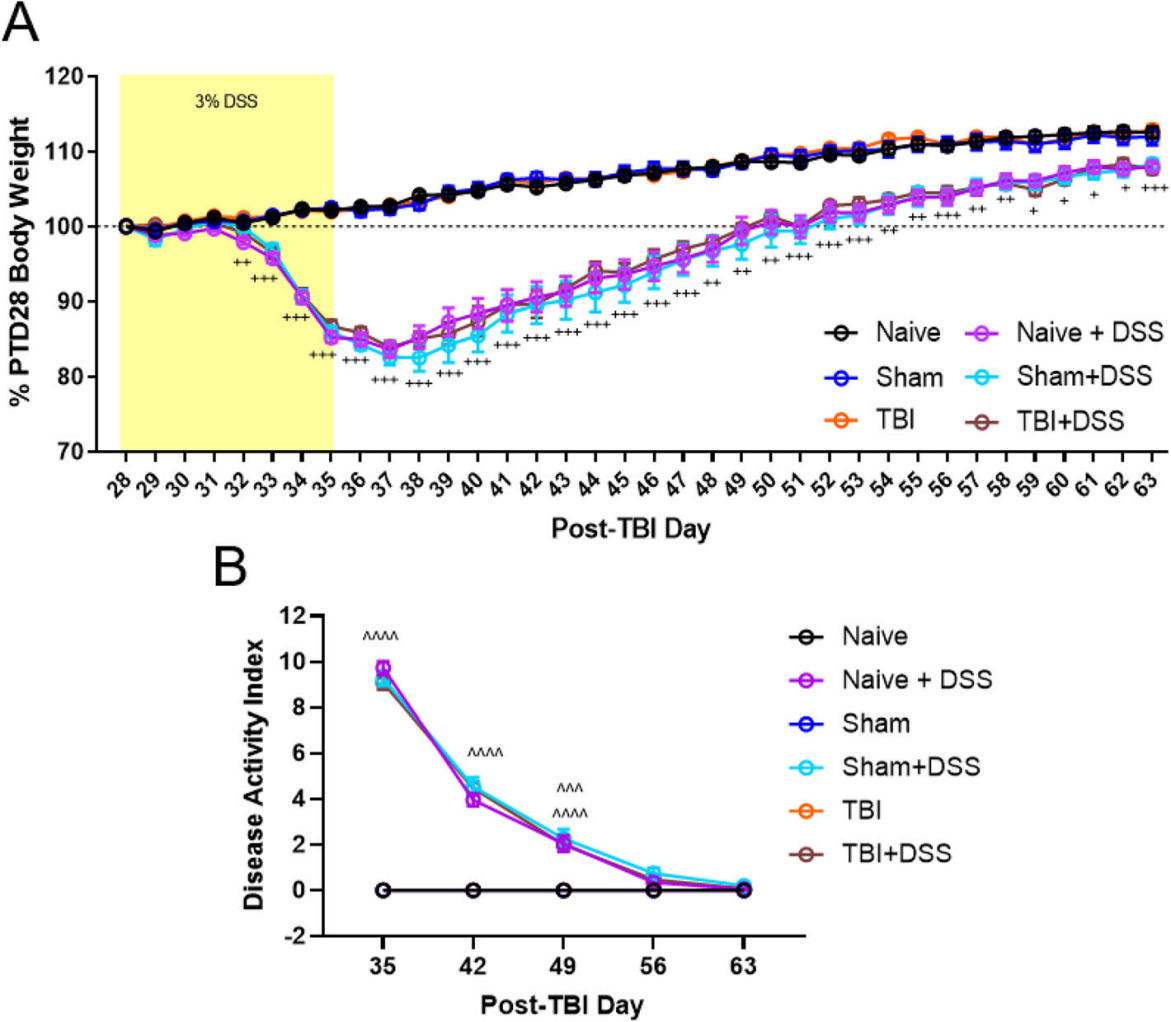
Prior TBI does not increase DSS disease severity or delay recovery. Prior craniotomy or TBI did not exacerbate the amount, or rate, of body weight lost upon DSS administration or regained during recovery **a**. Prior TBI or craniotomy did not increase disease activity index (DAI) scores at the end of the DSS injury phase (PTD35) in Sham+DSS and TBI + DSS mice compared to Naïve+DSS mice. DAI scores for DSS-treated mice returned to baseline values by the end of the third DSS recovery week (PTD 56) in a similar manner and rate, regardless of prior injury **b**. Data expressed as mean ± s.e.m (*cohort 3*, *n* = 15–21/group). **a** + *p* < 0.05 to *p* < 0.0001, ++ *p* < 0.01 to *p* < 0.0001, +++ *p* < 0.001 to *p* < 0.0001 vs water-treated counterparts; for specific *p* values see Supplemental Table 1. **b** ^^^^ *p* < 0.0001 Naïve vs Naïve+DSS, Sham vs Sham+DSS, TBI vs TBI + DSS, ^^^ *p* < 0.001 Naïve vs Naïve+DSS, Sham vs Sham+DSS

We assessed common indices of DSS injury severity and recovery throughout the study including body weight loss, disease activity index score (DAI), and morphometric changes in the colon. DSS is known to cause a significant loss in body weight in mice, up to 20% at the dose administered (3%) [30, 53]. Body weight loss upon DSS administration was calculated relative to body weight just prior to receiving DSS on PTD28. Naïve, Sham, and TBI mice administered DSS all lost a similar amount of weight, at a similar rate, peaking at PTD37 (TBI × DSS × Time Effect: F_(175, 3604)_ = 20.57, *p* < 0.0001; Naïve+DSS: 83.85 ± 1.08% (mean ± s.e.m.), Sham+DSS: 82.61 ± 1.07%, TBI + DSS: 83.91 ± 0.85%, Fig. 2a). All DSS-treated mice recovered their body weights at a similar rate as well, returning to PTD28 levels by the end of the second DSS recovery week (Naïve+DSS: 99.4 ± 1.83%, Sham+DSS: 97.63 ± 2.03%, TBI + DSS: 99.69 ± 0.71%). By the end of the fourth DSS recovery week, all DSS mice had regained similar amounts of weight (Naïve+DSS: 107.97 ± 0.67%, Sham+DSS: 108.35 ± 0.67%, TBI + DSS: 107.7 ± 0.74%).

DSS injury severity and recovery were also monitored by DAI scores. No differences were observed between any of the DSS-treated mice in their DAI scores throughout the study (DSS effect: F_(20,412)_ = 196.2, *p* < 0.0001; Fig. 2b). At the end of the DSS injury phase, all DSS-treated mice had equally elevated DAI scores (Naïve+DSS: 9.7 ± 0.3, Sham+DSS: 9.3 ± 0.3, TBI + DSS: 9.1 ± 0.3). DAI scores were similarly reduced within 1 week upon removal of DSS from the drinking water (Naïve+DSS: 4.0 ± 0.3, Sham+DSS: 4.5 ± 0.4, TBI + DSS: 4.5 ± 0.3), and returned to baseline levels by the end of the third DSS recovery week.

DSS is known to cause distinct morphometric changes in the colon [38]. As expected, DSS administration resulted in a significant shortening of the colon, increase in colon weight/body weight, and decrease in cecum weight/body weight at the end of the DSS injury phase compared to water-treated counterparts (Supplement Table 1). The observed decrease in colon length at this time point was similar between DSS-treated mice (*cohort 1*, DSS effect: F_(1, 58)_ = 31.31, *p* < 0.0001). By the end of the fourth week of the DSS recovery phase, colon morphology in DSS-treated mice was comparable to their water-treated counterparts (*cohort 3*, DSS effect: F_(1, 103)_ = 1.007, *p* = 0.3179), with no significant differences in colon length between the DSS mice (Supplement Table 1). Colon weight/body weight ratios increased equally in all DSS mice at the end of the DSS injury phase (*cohort 1*, DSS effect: F_(1, 58)_ = 55.22, *p* < 0.0001; Supplement Table 1). These increases in colon weight/body weight ratio improved over time, decreasing by the end of the fourth recovery week but still remained elevated compared to non-DSS mice (*cohort 3*, DSS effect: F_(1, 103)_ = 58.23, *p* < 0.0001; Supplement Table 1), with no observed differences between the DSS groups. Cecum weight/body weight ratios were reduced by DSS administration in all DSS mice by the end of the DSS injury phase (*cohort 1*, DSS effect: F_(1, 58)_ = 105.6, *p* < 0.0001), through the first week of the DSS recovery phase (*cohort 2*, DSS effect: F_(1, 86)_ = 172.5, *p* < 0.0001), to the fourth DSS recovery week where values returned closer to those observed in non-DSS-treated mice (*cohort 3*, DSS effect: F_(1, 103)_ = 18.89, *p* < 0.0001; Supplement Table 1).

Daily measuring of liquid intake was performed to monitor consistency of DSS consumption in mice. All mice administered DSS exhibited a similar decrease in water intake after day 3 of treatment that associated with weight loss, an index of disease severity (TBI × DSS × Time Effect: F_(170,3502)_ = 11.55, *p* < 0.0001; Supplemental Figure 1). Water intake returned to control levels upon removal of DSS from the drinking water consistent with initiation of the healing phase (Supplemental Figure 1). Importantly, as there were no observed differences in liquid intake between the DSS-treated mice; this suggests all observed changes in intestinal pathology, neurobehavior, and neuropathology are not due to variations in the amount of DSS consumed by mice. Combined, these data demonstrate that prior craniotomy or TBI do not affect the severity of injury induced by a chemical colitis challenge or the ability of the colon to respond to, or recover from, such a challenge.

### Intestinal inflammation exacerbates deficits in fine motor coordination in chronic TBI mice and induces deficits in fine motor coordination in Sham-injured mice

**Fig. 3:**
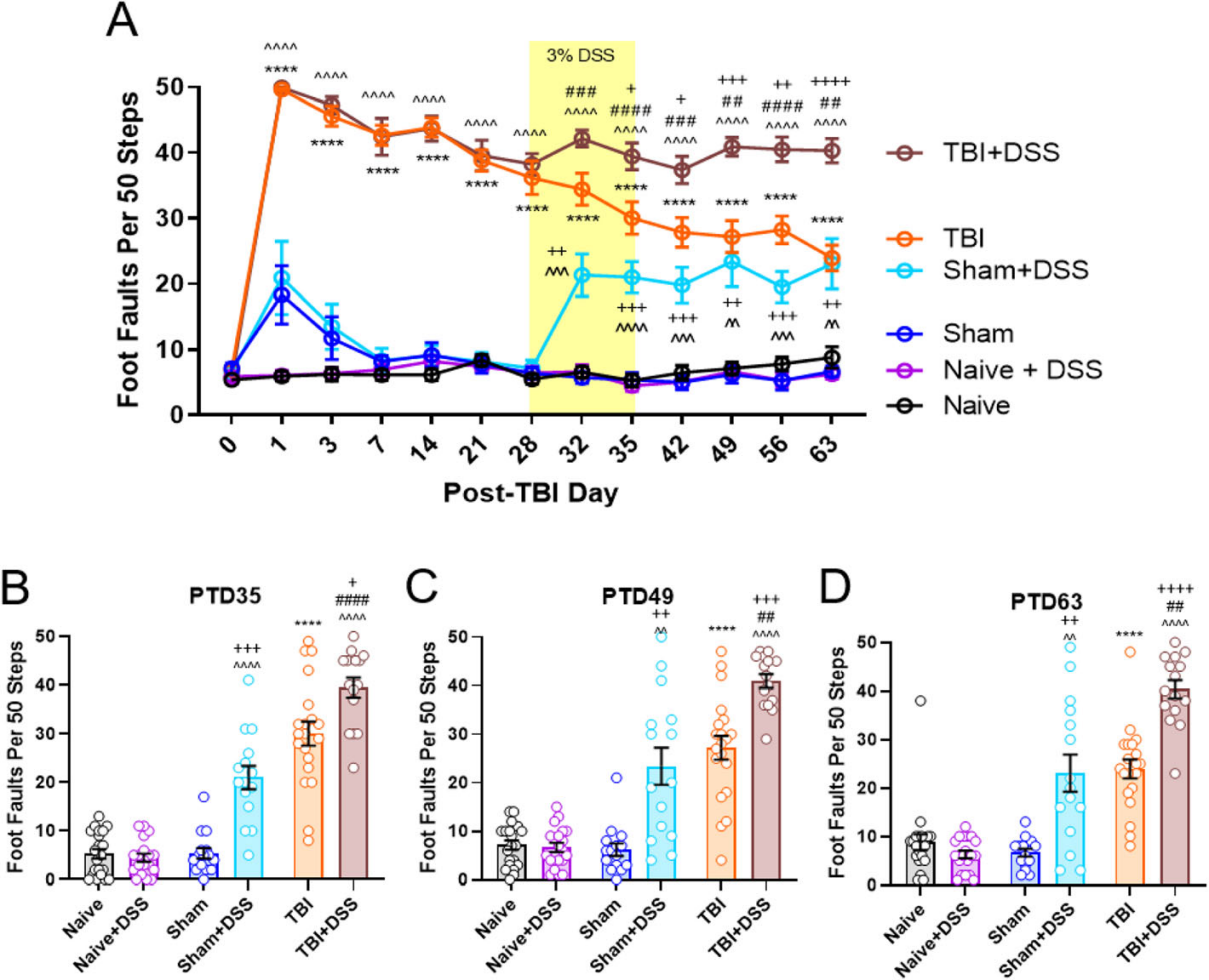
Intestinal inflammation during chronic TBI induces and exacerbates impairments in fine motor coordination. DSS administration induced increases in foot faults in Sham- and TBI mice beginning during the DSS injury phase (PTD32) and persisting through the end of the fourth week of the recovery phase (PTD63) **a–d**. Foot faults at specific time points within the study; PTD35 **b**, PTD49 **c**, and PTD63 **d**. No persistent impairments are observed in Sham-injured mice not administered DSS (Sham) or in Naïve mice administered DSS (Naïve+DSS). Data expressed as mean ± s.e.m (*n* = 15–21/group). PTD 1-28: **** *p* < 0.0001 TBI vs Naïve/Sham, ++++ *p* < 0.0001 TBI + DSS vs Naïve+DSS/Sham+DSS; PTD 32-63: **** *p* < 0.0001 TBI vs Naïve/Sham, ^^ *p* < 0.01 vs Naïve+DSS, ^^^ *p* < 0.001 vs Naïve+DSS, ^^^^ *p* < 0.0001 vs Naïve+DSS, + *p* < 0.05 vs Sham or TBI, ++ *p* < 0.01 vs Sham or TBI, +++ *p* < 0.001 vs Sham or TBI, ++++ *p* < 0.0001 vs Sham or TBI, ## *p* < 0.01 vs Sham+DSS, ### *p* < 0.001 vs Sham+DSS, #### *p* < 0.0001 vs Sham+DSS

TBI results in sustained deficits in fine motor coordination in the beam walk task [54]. Prior to initiation of intestinal inflammation, TBI mice exhibited a significant increase in foot faults beginning on the first day postinjury and persisting through the 63rd day postinjury (TBI × DSS × Time: F_(60,1186)_ = 21.73, *p* < 0.0001; Fig. 3a; Supplemental Table 2). On post-TBI day (PTD) 28, TBI mice had significantly more foot faults (ff), 37 ± 1 (mean ± s.e.m.), compared to Sham (7 ± 1, *p* < 0.0001) and Naïve mice (7 ± 1, *p* < 0.0001). No significant differences were observed between Sham and Naïve mice. Within 4 days of DSS administration (PTD32), foot faults increased in TBI + DSS (42 ± 1) compared to TBI mice (34 ± 2, *p* = 0.0815), reaching significance by the end of the DSS injury phase (PTD35, 30 ± 3 ff TBI vs 39 ± 2 ff TBI + DSS, *p* < 0.0454; Fig. 3a, b). This increase in foot faults in TBI + DSS vs TBI mice was sustained through the first (PTD42, 28 ± 2 ff TBI vs 37 ± 2 ff TBI + DSS, *p* = 0.0419; Fig. 3a), second (PTD49, 27 ± 2 ff TBI vs 41 ± 1 ff TBI + DSS, *p* = 0.0005; Fig. 3a, c), third (PTD56, 28 ± 2 ff TBI vs 41 ± 2 ff TBI + DSS, *p* = 0.0017; Fig. 3a), and fourth DSS recovery weeks (PTD63, 24 ± 2 ff TBI vs 40 ± 2 ff TBI + DSS, *p* < 0.0001; Fig. 3a, d). Remarkably, DSS administration also resulted in a significant increase in foot faults in Sham+DSS mice compared to their water-treated Sham counterparts and Naïve+DSS mice. This increase was observed within 4 days of the onset of DSS administration (PTD32, 21 ± 3 ff Sham+DSS vs 6 ± 1 ff Sham, 7 ± 1 ff Naïve+DSS; *p* = 0.0030 vs Sham, *p* = 0.0053 vs Naïve+DSS; Fig. 3a) and persisted through the end of the DSS injury phase (PTD35, 21 ± 2 ff Sham+DSS vs 5 ± 1 ff Sham, 4 ± 1 ff Naive+DSS; *p* = 0.0001 vs Sham, *p* < 0.0001 vs Naïve+DSS; Fig. 3a, b). Impaired motor coordination in Sham+DSS mice also extended through the end of the second (PTD49, 23 ± 4 ff Sham+DSS vs 6 ± 1 ff Sham, 7 ± 1 ff Naive+DSS; *p* = 0.0061 vs Sham, *p* = 0.0072 vs Naïve+DSS; Fig. 3a, c) and fourth week (PTD63, 23 ± 4 ff Sham+DSS vs 7 ± 1 ff Sham, 6 ± 1 ff Naïve+DSS; *p* = 0.0080 vs Sham, *p* = 0.0066 vs Naïve+DSS; Fig. 3a, d) of the DSS recovery phase. Importantly, no impairments were observed in Sham-injured mice prior to DSS administration (Fig. 3a) or in Naïve mice administered DSS (Naïve+DSS) (Fig. 3a-d).

No significant impairments in gross motor function, as assessed through locomotor activity during NOR habituation testing (Table 1) or swim speeds during MWM probe trial testing (Fig. 5d), were induced by DSS. Following TBI, and prior to DSS administration, TBI mice exhibited hyperactivity in terms of significantly increased distance traveled (Injury Effect: F_(2, 107)_ = 9.473, *p* = 0.0002; TBI:37.911 ± 1.954 meters (m), Sham:32.679 ± 0.856 m, Naïve:30.461 ± 0.859 m; *p* = 0.0256 TBI vs Sham, *p* = 0.0001 TBI vs Naïve; Table 1) and speed (Injury Effect: F_(2, 107)_ = 2.432, *p* = 0.0002; TBI:0.031 ± .003 meters/second (m/s), Sham:0.055 ± 0.001 m/s, Naïve:0.051 ± 0.001 m/s, *p* = 0.0390 vs Sham, *p* = 0.0001 vs Naïve; Table 1) compared to Sham and Naïve mice. During DSS administration and through the fourth week of the DSS recovery phase, there were no significant differences between any of the experimental groups in terms of the distance traveled or average speed (Table 1). Average swim speeds measured during the probe trial were also similar between all experimental groups (TBI × DSS effect: F_(2, 100)_ = 0.3018, *p* = 0.7420; Fig. 5f). Taken together, these data demonstrate that neither DSS-induced colitis nor craniotomy alone alters gross motor function and that the changes induced by TBI alone or intestinal inflammation in Sham and TBI mice are confined to fine motor coordination, with gross motor function remaining intact.

**Table 1:**
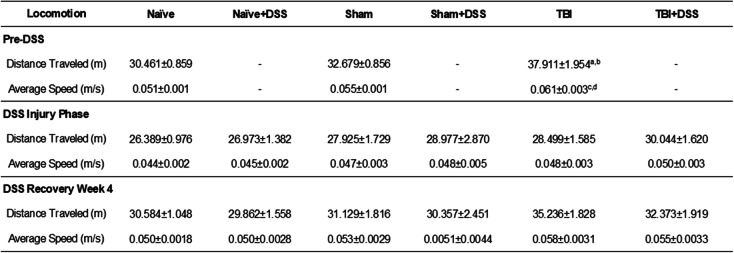
General locomotion is not altered by intestinal inflammation during chronic TBI. Distance traveled and average speed was measured during NOR habituation testing carried out prior to, during, and following DSS administration. All experimental groups performed at similar levels indicating no significant impairment of gross motor function. Data expressed as mean ± s.e.m (*n* = 31–42 per group pre-DSS; *n* = 15–21 per group DSS injury/recovery phases). a = *p* = 0.0001 vs Naïve, b = *p* = 0.0256 vs Sham, c = *p* = 0.0001 vs Naïve, d = *p* = 0.0390 vs Sham. Abbreviations: *m* meters, *m/s* meters/second

### Intestinal inflammation impairs cognition in Sham-injured mice

**Fig. 4:**
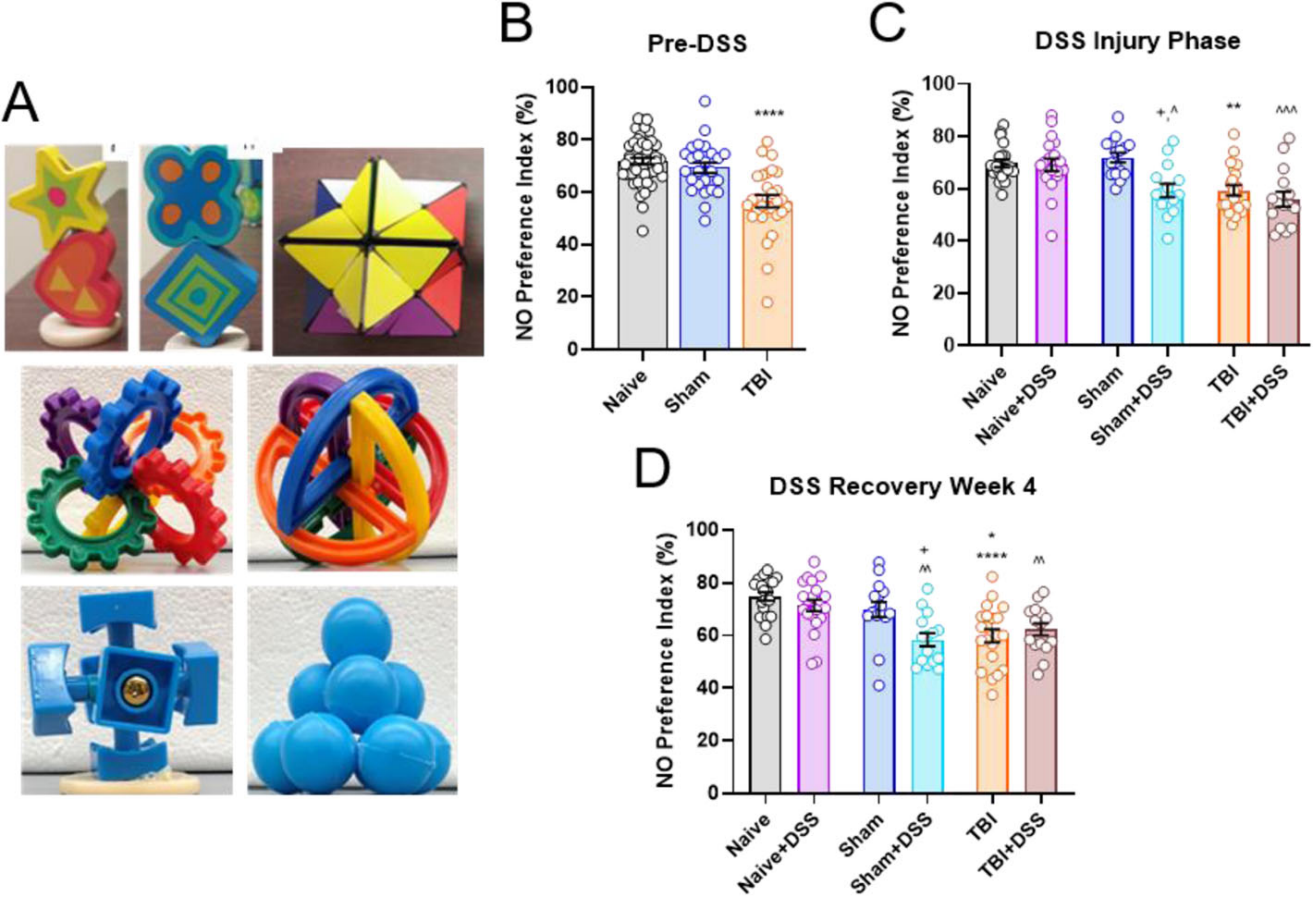
Intestinal inflammation induces persistent impairments in declarative memory in Sham-injured mice. Objects used during NOR testing **a**. TBI mice spent less time exploring the novel object (NO) prior to the onset of DSS administration (PTD25-27). No significant changes in NO PI were observed in Sham-injured mice **b**. Sham+DSS mice exhibited a significant reduction in the time spent with the NO compared to Sham mice, beginning during the DSS injury phase **c** and persisting through the fourth week of the recovery phase **d**. Further reduction in time spent with the NO in TBI + DSS vs TBI mice was not observed **c**, **d**. No significant changes were observed in Naïve+DSS mice. Data expressed as mean ± s.e.m (*n* = 31–42/group Pre-DSS; *n* = 15–21/group DSS injury/recovery phases). **b** **** *p* < 0.0001 vs Naïve/Sham; **c**,**d** * *p* < 0.05 vs Sham ,**p < 0.01 vs Naïve/Sham, **** *p* < 0.0001 vs Naïve, ^ *p* < 0.05 vs Naïve+DSS, ^^ *p* < 0.01 vs Naïve+DSS, ^^^ *p* < 0.001 vs Naïve+DSS, + *p* < 0.05 vs Sham

To examine the effects of intestinal inflammation on cognitive function, NOR and MWM were carried out to assess changes in declarative memory and hippocampal-dependent spatial learning and memory, respectively. NOR testing was carried out prior to, during, and following DSS administration. At all time points of familiarization stage of testing, there were no significant differences between any of the experimental groups in terms of how much time was spent with either of the two objects presented, confirming the absence of an overarching side bias (Supplemental Figure 2). Naïve mice typically spend ~ 70% of their time exploring the novel over the familiar object, with 50% being chance. Therefore, a decrease in novel object (NO) preference index (PI) indicates a disruption in familiar object memory and declarative memory overall. During NO testing, TBI mice spent significantly less time exploring the NO versus the familiar object (TBI effect: F_(2, 102)_ = 21.32, *p* < 0.0001; mean ± s.e.m.; TBI: 56.51 ± 2.37% NO PI, Naïve:71.91 ± 1.26% NO PI, Sham: 69.30 ± 1.95% NO PI, *p* < 0.0001 vs Naïve and Sham; Fig. 4b). DSS administration did not further decrease NO PI in TBI + DSS mice during either the DSS injury (DSS effect: F_(1, 102)_ = 8.466, *p* = 0.0045; 59.34 ± 2.00% TBI vs 56.08 ± 2.71% TBI + DSS; Fig. 4c) or recovery phases (DSS effect: F_(1, 102)_ = 4.801, *p* = 0.0307; 59.87 ± 2.52% TBI vs 62.29 ± 2.35% TBI + DSS; Fig. 4d). DSS administration did significantly reduce the amount of time Sham+DSS mice spent with the NO (59.34 ± 2.69% NO PI) compared to Sham (71.85 ± 1.60% NO PI, *p* = 0.0101) and Naïve+DSS (69.13 ± 1.69% NO PI, *p* = 0.0291) mice during the DSS injury phase (Fig. 4c). This significant decrease in NO PI in Sham+DSS mice was comparable to TBI mice (59.34 ± 2.00%) and was sustained through the end of the fourth recovery week (DSS effect: F_(1, 102)_ = 4.801, *p* = 0.0307; Sham+DSS: 58.31 ± 2.47%, Sham: 69.89 ± 2.85%, Naïve+DSS:71.38 ± 2.14, *p* = 0.0185 vs Sham, *p* = 0.0023 vs Naïve+DSS; Fig. 4d). No deficits were observed in Sham-injured mice prior to the onset of DSS administration (69.30 ± 1.95%) or in Naïve mice administered DSS. No significant differences were observed between any experimental group at any time point of testing in terms of total amount of time spent exploring the objects presented (Supplemental Figure 3).

**Fig. 5:**
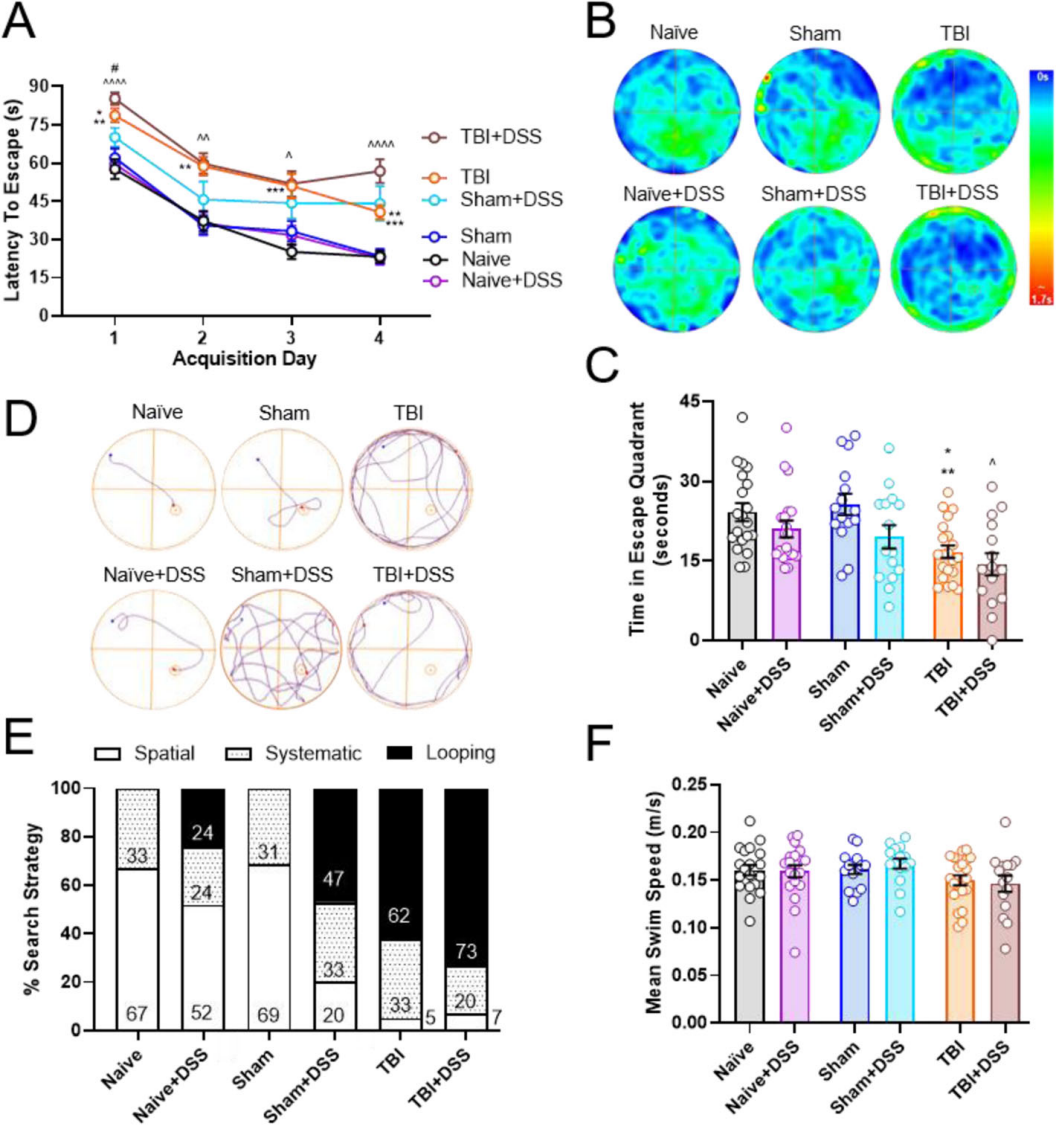
Deficits in spatial learning and memory are observed in Sham-injured mice subjected to intestinal inflammation. TBI and TBI + DSS mice required significantly more time to locate the hidden platform during acquisition training carried in the first DSS recovery week compared to Naïve/Sham and Naïve+DSS/Sham+DSS mice, respectively. No significant changes in escape latency were observed between these two groups. Sham+DSS mice exhibited increased escape latency compared to Sham counterparts, but this increase did not reach significance. No differences were observed in escape latencies for Naïve, Sham or Naïve+DSS mice **a**. Representative heat maps of time spent in the escape quadrant during probe trial **b**. TBI and TBI + DSS mice spent significantly less time in the escape quadrant during probe trial compared to Naïve/Sham and Naïve+DSS mice, respectively. No significant differences were found in the time spent in the target quadrant during probe trial between Naïve, Naïve+DSS, Sham and Sham+DSS mice **c**. Representative track plots of swim patterns during probe trial **d**. Assessment of search strategy employed during probe trial showed that Sham+DSS mice increasingly used the looping strategy, and decreasingly used the spatial strategy, compared Sham mice. Increased reliance on looping search strategies in Naïve+DSS and TBI + DSS mice compared to Naïve or TBI mice, respectively, did not reach significance **e**. Swim speeds were similar between all mice during probe trial **f**. Data expressed as mean ± s.e.m (*n* = 15–21/group). **a** AD1: * *p* < 0.05 vs Sham, ** *p* < 0.01 Naïve, ++++ *p* < 0.0001 vs Naïve+DSS; AD2:*** vs Naïve/Sham, ++ *p* < 0.01 vs Naïve+DSS; AD3; *** *p* < 0.001 vs Naïve+DSS, +*p* < 0.05 vs Naïve+DSS; AD4: 88 *p* < 0.01 vs Sham, *** *p* < 0.001 vs Naïve, ++++ *p* < 0.0001 vs Naïve+DSS. **b** * *p* < 0.05 vs Naïve, ** *p* < 0.01 vs Sham, ^ *p* < 0.05 vs Naïve+DSS

MWM testing to assess hippocampal-dependent spatial learning and memory was performed during the first recovery week (PTD 38-42). Both TBI and TBI + DSS mice exhibited increased latencies to locate the platform compared to Naïve/Sham and Naïve+DSS mice, respectively (TBI × DSS × Time: F_(15,300)_ = 1.022, *p* = 0.4317; AD1-4: *p* < 0.05 to *p* < 0.0001 TBI + DSS vs Naïve+DSS; *p* < 0.01 vs *p* < 0.001 vs TBI vs Naïve; Fig. 5a). Although latency to escape for Sham+DSS mice increased, this increase was not significance (DSS × Time: F_(5, 100)_ = 17.26, *p* < 0.0001; AD1-4: *p* = 0.6277, *p* = 0.7983, *p* = 0.6479, *p* = 0.0977; Fig. 5a). While TBI (16.7 ± 1.2 s) and TBI + DSS (14.4 ± 2.1 s) mice spent significantly less time in the target quadrant compared to Naïve/Sham (Naïve: 24.0 ± 1.6 s, Sham: 24.7 ± 2.1 s) and Naïve+DSS mice (20.15 ± 2.2 s), respectively (TBI × DSS effect: F_(2, 100)_ = 0.5710, *p* = 0.5668; *p* = 0.0186 TBI vs Naïve; *p* = 0.0069 TBI vs Sham; *p* = 0.0257 TBI + DSS vs Naïve+DSS), no significant decreases were observed in the Sham+DSS mice (19.5 ± 2.2 s, Fig. 5b, c). Additionally, no significant differences were found between Naïve and Naïve+DSS mice in the amount of time spent in the target quadrant (Fig. 5b, c). Search strategy employed by the mice to locate the hidden platform was assessed by analyzing the swim pattern for each mouse. Based on swim patterns, mice were assigned to one of three categories (spatial, non-spatial systematic, or repetitive looping) as previously described [43]. Water-treated TBI mice predominately used a looping search strategy (63%, *χ*^2^ = 24.27, *p* < 0.0001 vs Naïve; *χ*^2^ = 21.38, *p* < 0.0001 vs Sham), whereas water-treated Sham-injured and Naïve mice primarily used a spatial search strategy; 67% and 69%, respectively. Use of the repetitive looping search strategy was not further significantly increased in TBI + DSS (72%) compared to TBI mice (*χ*^2^ = 0.7886, *p* < 0.6742 vs TBI + DSS). Administration of DSS to Sham-injured mice (Sham+DSS) resulted in a shift to predominately using the looping search strategy (47%) as opposed to the spatial search strategy (69%) employed by their Sham counterparts (*χ*^2^ = 11.55, *p* < 0.0031, Fig. 5d, e). While Naïve+DSS mice utilized the looping search strategy whereas Naïve mice did not, this did not reach significance (24% Naïve+DSS vs 0% Naïve, *χ*^2^ = 5.693, *p* < 0.0580). Swim speeds were similar among all experimental groups, indicating that any differences observed during acquisition testing and probe trial were due to cognitive changes independent of general motor function (TBI × DSS effect: F_(2, 100)_ = 0.3018, *p* = 0.7420; Fig. 5f). These results show that intestinal inflammation following craniotomy and TBI increased reliance on a less efficient spatial search strategy, indicating an impairment in spatial navigation, learning, and memory. Moreover, in concordance with the NOR results, these data suggest that a TBI ceiling effect may be present, obscuring any additive effects induced by intestinal inflammation. Finally, these data show that intestinal inflammation may have differential effects on cognition, inducing more pronounced deficits in declarative memory then spatial learning and memory.

### Social behavior is negatively affected by intestinal inflammation during chronic TBI in Sham-injured and TBI mice

**Fig. 6:**
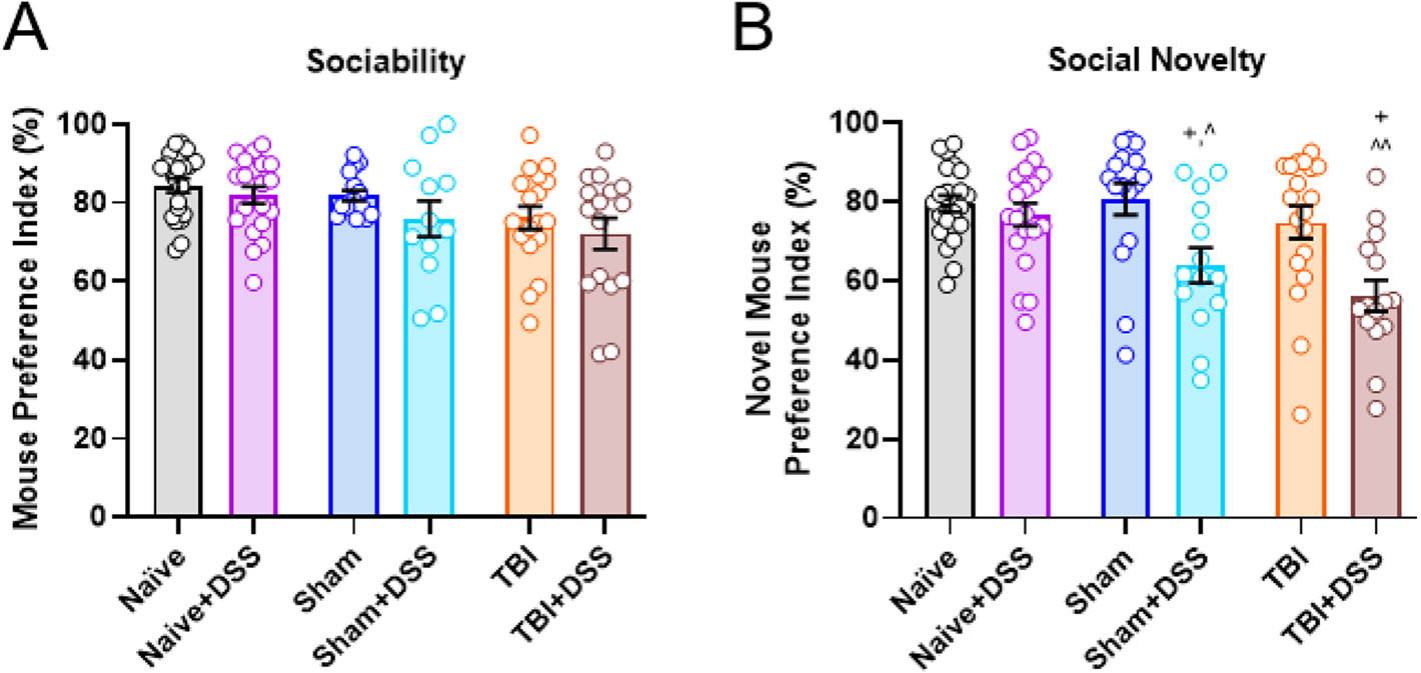
Deficits in social behavior are induced by intestinal inflammation in Sham-injured and TBI mice. Neither TBI nor intestinal inflammation significantly altered sociability in mice **a**. Intestinal inflammation following craniotomy and TBI resulted in an impairment in social recognition and memory with both Sham+DSS and TBI + DSS mice spending significantly less time with the novel stimulus mouse over the familiar stimulus mouse compared to Sham and TBI mice, respectively **b**. Data expressed as mean ± s.e.m (*n* = 12–21/group). ^ *p* < 0.05 vs Naïve+DSS, ^^ *p* < 0.01 vs Naïve+DSS, + *p* < 0.05 vs Sham or TBI

TBI and DSS-induced colitis are reported to alter social behavior in rodents [55, 56]. We next assessed the effect of intestinal inflammation during chronic TBI on social behavior using Crawley’s three-chamber social approach task (SA) 3 weeks after the onset of intestinal inflammation (PTD51-54). No changes in sociability (stage 2) between the experimental groups were observed (TBI × DSS Effect: F_(2, 97)_ = 0.1638, *p* = 0.8491; Fig. 6a). However, DSS administration decreased the PI for the novel stimulus mouse over the familiar stimulus mouse (stage 3) in Sham and TBI mice when compared to their water-treated counterparts (DSS effect: F_(1, 97)_ = 18.39, *p* < 0.0001; Sham: 80.76 ± 4.02% PI vs 62.21 ± 4.75% PI Sham+DSS, *p* = 0.0187; TBI: 74.27 ± 2.23% PI vs TBI + DSS: 57.34 ± 3.87% PI, *p* = 0.0163; Fig. 6b). Novel mouse PI was similar between Naïve and Naïve+DSS mice, indicating intestinal inflammation alone does not alter social behavior (Naïve: 79.84 ± 2.23% vs Naïve+DSS: 76.99 ± 3.10%, *p* = 0.9867). Within both stages of SA, no significant differences were found between experimental groups in the total amount of time spent exploring the stimulus mice/object (Stage 2: TBI × DSS effect: F_(2, 97)_ = 1.74, *p* = 0.1809; Stage 3: TBI × DSS effect: F_(2, 97)_ = 0.3800, *p* = 0.6484; Supplemental Figure 4B). These data suggest that deficits in social behavior induced by intestinal inflammation during chronic TBI affect primarily social recognition and memory, in agreement with deficits in declarative memory, without significantly altering an animal’s willingness to socialize with other animals (sociability).

### Sham-injured and TBI mice exhibit increases in anxiety-like and repetitive-like behavior following intestinal inflammation

**Fig. 7:**
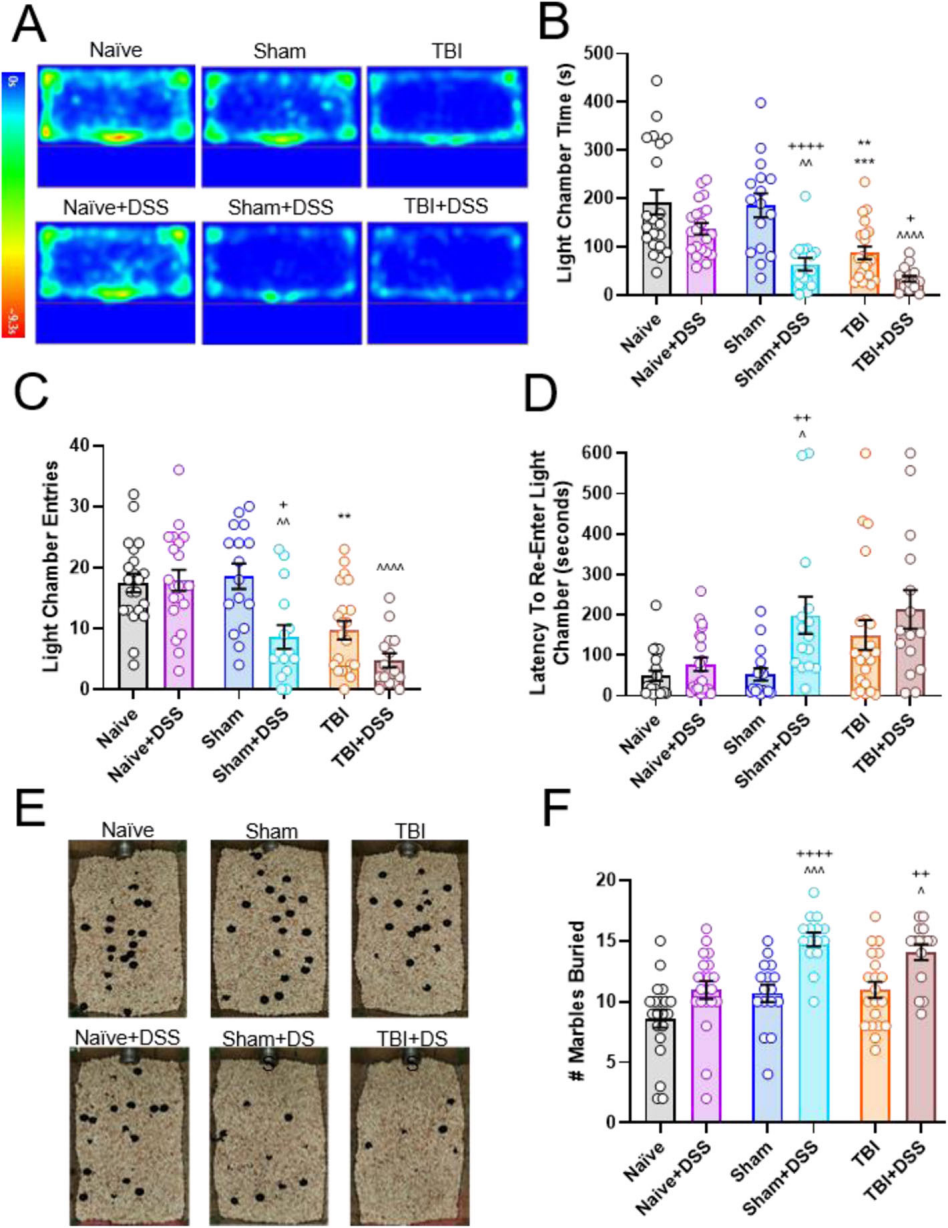
Anxiety- and repetitive-like behavior is increased following intestinal inflammation in Sham-injured and TBI mice. Representative heatmaps of mouse presence in the light chamber during light-dark box testing carried out in the third DSS recovery week **a**. TBI mice spent less time in the light chamber compared to Naïve and Sham mice. This was further decreased with DSS administration (TBI + DSS). DSS administration also decreased time spent in the light chamber in Sham+DSS mice compared to Sham mice **b**. TBI and TBI + DSS mice had fewer entries into the light chamber compared to Naïve/Sham and Naïve+DSS mice, respectively. Light chamber entries were also decreased in Sham+DSS mice compared to Sham mice **c**. TBI and TBI + DSS mice exhibited increased latency to re-enter the light chamber from the dark chamber compared to Naïve/Sham and Naïve+DSS/Sham+DSS mice, respectively. DSS administration significantly increased light chamber re-entry latency in Sham+DSS compared to Sham mice **d**. Representative images from marble burying conducted during the third DSS recovery week **e**. Both Sham+DSS and TBI + DSS buried significantly more marbles than their water-treated counterparts **f**. Data expressed as mean ± s.e.m (*n* = 15–21/group). **b** ** *p* = 0.01 vs Sham, *** *p* = 0.001 vs Naïve, ^^ *p* < 0.01 vs Naïve+DSS, ^^^ *p* < 0.0001 vs Naïve+DSS, + *p* < 0.05 vs TBI, ++++ *p* < 0.0001 vs Sham. **c**,**d** ** *p* = 0.01 vs Naïve/Sham, ^ *p* < 0.05 vs Naïve+DSS, ^^ *p* < 0.01 vs Naïve+DSS, ^^^ *p* < 0.0001 vs Naïve+DSS, + *p* < 0.05 vs Sham, ++ *p* < 0.01 vs Sham. **e** ^ *p* < 0.05 vs Naïve+DSS, ^^^ *p* < 0.001 vs Naïve+DSS, + *p* < 0.01 vs TBI, ++++ *p* < 0.0001 vs Sham

Both TBI and DSS-induced colitis have an impact on affective behavior [20, 56]. Light-dark box (LDB) and marble burying (MB) tasks were performed during the third DSS recovery week to assess anxiety-like behavior and repetitive-like/anxiety-like behavior, respectively. During LDB testing, TBI mice spent less time in the open, light chamber compared to Naïve and Sham mice (TBI effect: F_(2, 103)_ = 3.45, *p* < 0.0001; TBI: 87.75 ± 13.20s, Naïve: 192.19 ± 25.18 s, Sham:185.61 ± 24.49 s; *p* = 0.0003 vs Naïve, *p* = 0.0019 vs Sham; Fig. 7a, b). DSS administration further decreased the amount of time TBI + DSS mice spent in the light chamber compared to TBI mice (33.59 ± 6.26 s, DSS effect: F_(1, 103)_ = 34.45, *p* < 0.0001; *p* = 0.0260 vs TBI). Sham+DSS mice also exhibited a decrease in the amount of time spent in the light chamber compared to Sham mice (185.61 ± 24.49 s Sham vs 63.76 ± 13.44 s Sham+DSS, *p* < 0.0001). Naïve+DSS mice spent a similar amount of time in the light chamber compared to Naïve mice (137.03 ± 11.81 s, *p* = 0.4722 vs Naïve). Corresponding to decreases in time spent in the light chamber, TBI mice also had fewer entries into the light chamber compared to Naïve and Sham mice (TBI effect: F_(2, 103)_ = 20.42, *p* < 0.0001; TBI: 10 ± 2 entries, Naïve: 17 ± 2 entries, Sham:19 ± 2 entries; *p* < 0.0086 vs Naïve, *p* = 0.0043 vs Sham; Fig. 7c). TBI + DSS mice had fewer light chamber entries (5 ± 2 entries) but this did not reach significance compared to TBI mice (DSS effect: F_(1, 103)_ = 12.09, *p* = 0.0043; 5 ± 2 entries, *p* = 0.3195). Sham+DSS mice also had fewer entries into the light chamber than Sham mice (19 ± 2 entries Sham vs 9 ± 2 Sham+DSS, *p* = 0.0026). Light chamber entries were similar between Naïve and Naïve+DSS mice (17 ± 2 entries Naïve vs 18 ± 2 Naïve+DSS, *p* > 0.9999). Latency to re-enter the light chamber once in the dark chamber was also increased in Sham+DSS mice compared to Sham mice (DSS effect: F_(1, 103)_ = 13.35, *p* = .0004; 52.39 ± 15.44 s Sham vs 198.31 ± 46.28 s Sham+DSS, *p* = 0.0073, Fig. 7d). While both TBI and TBI + DSS had increased re-entry latencies compared to Naïve and Naïve+DSS mice, respectively, this did not reach significance (TBI: 149.78 ± 36.41 s, *p* < 0.0770 vs Naïve, TBI + DSS: 213.52 ± 47.84 s, *p* < 0.0590 vs Naïve+DSS). Re-entry latency was similar between Naïve and Naïve+DSS mice (49.03 ± 12.71 s, 77.55 ± 16.77 s, respectively).

MB has been used as a measure of both anxiety-like and perseverative behavior in rodents [46, 47]. In MB testing carried out during the third DSS recovery week, both Sham-injured and TBI mice administered DSS buried significantly more marbles compared to water-treated Sham and TBI mice (DSS effect: F_(1, 101)_ = 39.92, *p* < 0.0001; mean ± s.e.m.; 11 ± 1 marbles Sham vs 15 ± 1 Sham+DSS, *p* = 0.0026; 11 ± 1 marbles TBI vs 14 ± 1 marbles TBI + DSS, *p* = 0.0091; Fig. 7e, f). DSS did not significantly increase number of marbles buried in Naïve+DSS mice compared to Naïve mice (9 ± 1 marbles vs 11 ± 1 marbles, *p* = 0.1517). Taken together, these data indicate that DSS-induced colitis alone does not affect anxiety-like or perseverative behavior but does significantly increase anxiety-like and repetitive-like behavior in Sham-injured and TBI mice.

### Intestinal inflammation exacerbates TBI-associated hippocampal neurodegeneration and induces hippocampal neurodegeneration in Sham-injured mice

**Fig. 8:**
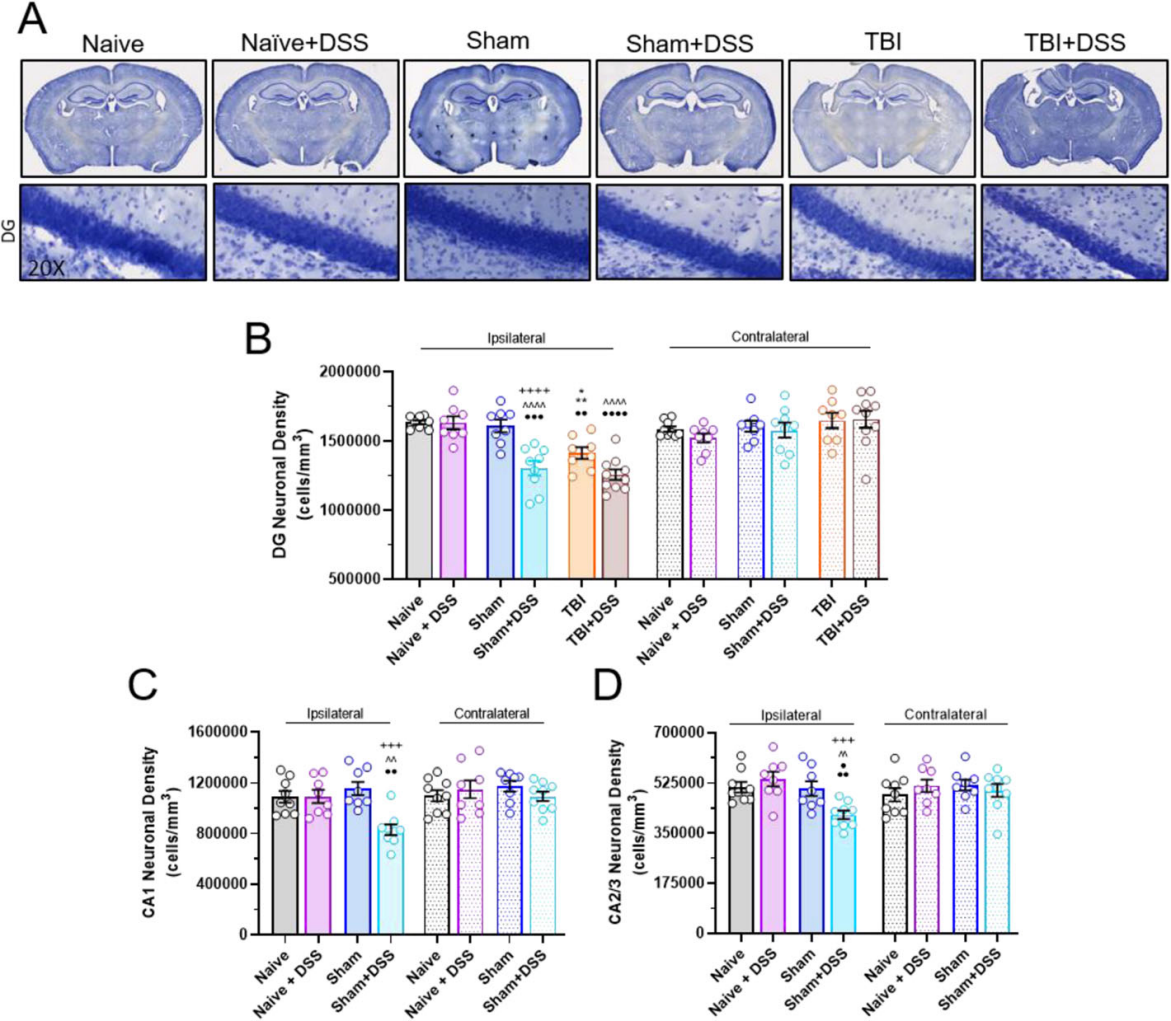
Hippocampal neurodegeneration is induced by intestinal inflammation in Sham-injured mice and exacerbated in TBI mice. One week following the cessation of DSS administration, ipsilateral hippocampal neuronal cell density was assessed by stereological quantification **b–d**. Representative images of cresyl violet stained brains **a**, (upper images) and neurons in the dentate gyrus (DG) region of the ipsilateral hippocampus **a**, (lower images, ×20 magnification) across all experimental groups. Quantification of neurons in the DG subregion revealed a significant increase in ipsilateral neuronal cell loss in the TBI + DSS compared to TBI mice. DSS administration also induced a significant loss in ipsilateral neurons in the DG in Sham+DSS compared to Sham mice **b**. A significant loss of ipsilateral neurons in the cornu ammonis (CA) 1 and CA2/3 hippocampal subregions was also observed in Sham+DSS mice compared to Sham mice. No significant changes in neuronal cell counts in the ipsilateral hippocampus of Naïve+DSS mice or in the contralateral hippocampus of all mice were observed **b**–**d**. Data expressed as mean ± s.e.m (*cohort 2*, *n* = 8–10/group). **b** * *p* < 0.0399 vs Sham ipsi, ** *p* < 0.0101 vs Naïve ipsi, ^^^^ *p* < 0.0001 vs Naïve+DSS ipsi, + *p* < 0.05 vs TBI ipsi, ++++ *p* < 0.0001 vs Sham ipsi, •• *p* < 0.05 vs TBI contra, ••• *p* < 0.001 vs Sham+DSS contra, •••• *p* < 0.0001 vs TBI + DSS contra. **c** ^^^^ *p <* 0.0001 vs Naïve+DSS ipsi, +++ *p* = 0.001 vs Sham ipsi, •• *p* < 0.05 vs Naïve+DSS/Sham+DSS contra. **d** ^^ *p =* 0.01 vs Naïve+DSS ipsi, +++ *p* = 0.001 vs Sham ipsi, • *p* < 0.05 vs Naïve+DSS contra, •• *p* < 0.05 vs Sham+DSS contra;. Abbreviations: contra, contralateral; CA, cornu ammonis; DG, dentate gyrus; ipsi, ipsilateral; mm, millimeter

To investigate the effect of intestinal inflammation during chronic TBI on neuropathological outcomes, cortical lesion volume and hippocampal neuronal cell loss were assessed at the end of the first week of the DSS recovery phase (PTD42-43). As previously reported, TBI resulted in a large cortical lesion in the ipsilateral cortex (mean ± s.e.m.; 8.16 ± 0.56 mm^3^; Fig. 8a). Administration of DSS to TBI mice (TBI + DSS) did not further exacerbate the volume of the cortical lesion (7.96 ± 0.51 mm^3^; *t*_(11)_ = 0.2564, *p* = 0.8024 vs TBI). As expected, TBI also resulted in a significant loss of neurons in the dentate gyrus (DG) subregion of the ipsilateral hippocampus (1.45 × 10^4^ ± 4.46 × 10^4^ cells/mm^3^) compared to Naïve and Sham mice (1.64 × 10^6^ ± 1.72 × 10^4^ cells/mm^3^; 1.63 × 10^6^ ± 4.58 × 10^4^ cells/mm^3^, respectively; TBI × DSS effect: F_(5, 92)_ = 3.573, *p* = 0.0054; *p* = 0.0101 vs Naïve, *p* = 0.0399 vs Sham; Fig. 8b). DSS-treated TBI mice exhibited a further increase in ipsilateral DG neuronal cell loss but did not reach significance (1.23 × 10^6^ ± 3.44 × 10^4^ cells/mm^3^, *p* = 0.0598 vs TBI). DSS administration also caused a significant loss of ipsilateral DG neurons in Sham+DSS compared to Sham mice (1.63 × 10^6^ ± 4.58 × 10^4^ cells/mm^3^ Sham+DSS, 1.29 × 10^6^ ± 6.54 × 10^4^ cells/mm^3^ Sham; *p* < 0.0001 vs Sham; Fig. 8b). Corresponding to the loss of neurons in the DG, Sham+DSS mice also had significantly reduced neuronal cell densities in the cornu ammonis (CA) 1 and CA2/3 subregions compared to Sham mice (CA1: TBI × DSS effect: F_(3, 60)_ = 5.912, *p* = 0.0013; 1.16 × 10^6^ ± 5.24 × 10^4^ cells/mm^3^ Sham vs 8.32 × 10^5^ ± 4.23 × 10^4^ cells/mm^3^ Sham+DSS; *p* = 0.0004 vs Sham; CA2/3: TBI × DSS effect, F_(3, 60)_ = 5.457, *p* = 0.0022; 5.06 × 10^5^ ± 2.55x10^4^ cells/mm^3^ Sham vs 4.14 × 10^5^ ± 1.45 × 10^4^ cells/mm^3^ Sham+DSS; *p* = 0.0425 vs Sham; Fig. 8c, d). No significant differences were observed between the Naïve and Naïve+DSS mice in any of the subregions examined (CA1: 1.09 × 10^6^ ± 4.56 × 10^4^ cells/mm^3^ Naïve vs 1.09 × 10^6^ ± 5.18 × 10^4^ cells/mm^3^ Naïve+DSS, *p* > 0.9999; CA2/3: 5.11 × 10^5^ ± 2.05 × 10^4^ cells/mm^3^ Naïve vs 5.39 × 10^5^ ± 2.55 × 10^4^ cells/mm^3^ Naïve+DSS, *p* ≥ 0.9864; DG: 5.11 × 10^5^ ± 2.05 × 10^4^ cells/mm^3^ Naïve vs 5.39 × 10^5^ ± 2.55 × 10^4^ cells/mm^3^ Naïve+DSS, *p* > 0.9999). Additionally, all observed changes in neuronal cell densities were confined to the ipsilateral hippocampus, with no significant changes found between experimental groups in the contralateral hippocampus (Fig. 8c-e). These results demonstrate that intestinal inflammation during chronic TBI or craniotomy induces, or exacerbates, hippocampal neurodegeneration, in concurrence with observed impairments in neurobehavior, suggesting physical alteration of neural pathways involved in cognition.

### Intestinal inflammation worsens TBI-associated microglial activation and induces microglial activation in Sham-injured mice

**Fig. 9:**
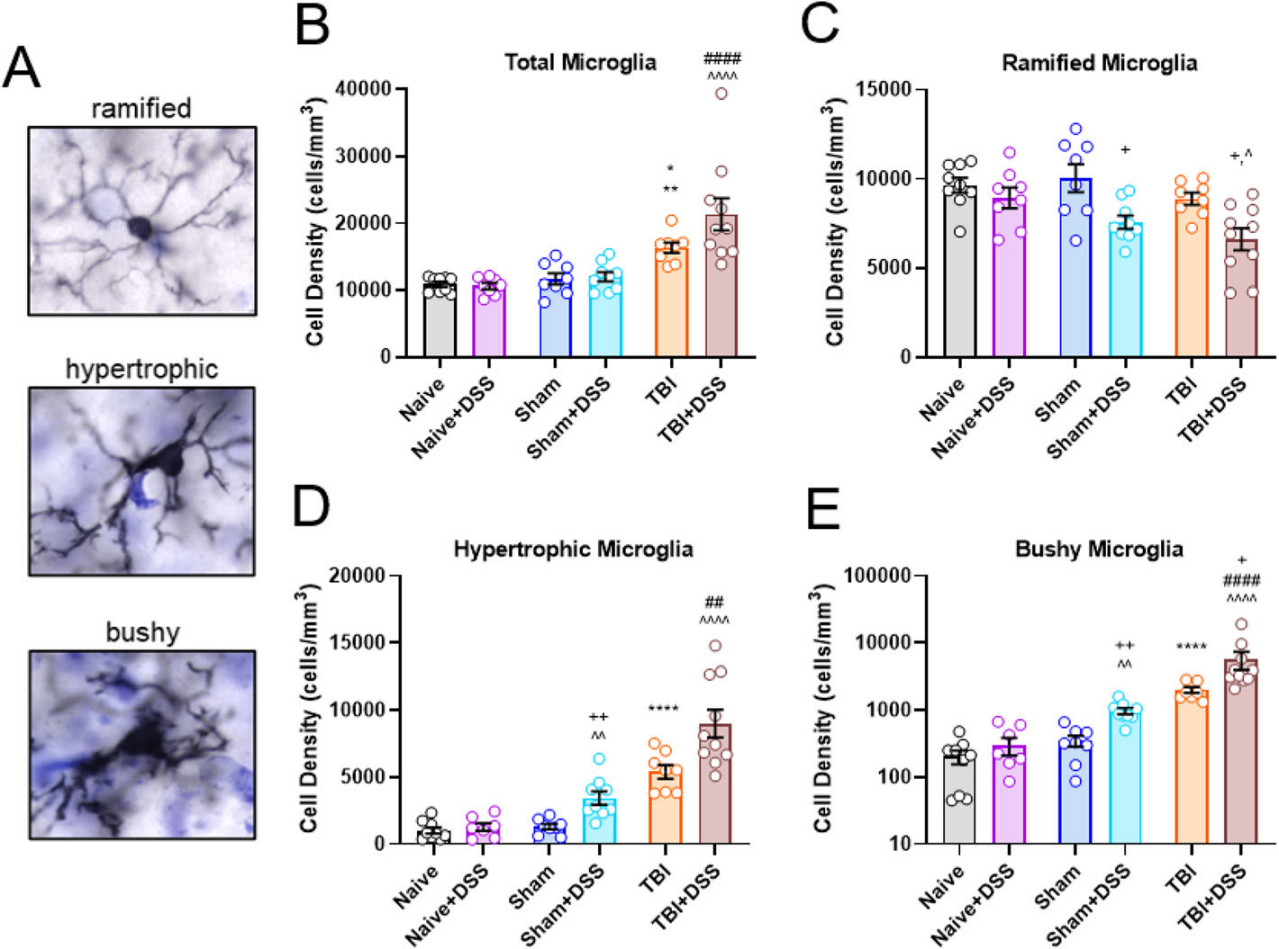
Microglial morphology is altered by intestinal inflammation during chronic TBI. Representative images of Iba1+ microglia displaying ramified, hypertrophic, and bushy morphologies **a**. TBI significantly increased the number of total **b**, hypertrophic **d**, and bushy **e** of Iba1+ microglia in the ipsilateral hippocampus compared to Naïve and Sham mice. DSS administration resulted in a further increase in bushy Iba1+ microglia **e**, while decreasing the number of ramified Iba1+ microglia **c** in the ipsilateral hippocampus of TBI + DSS mice compared to TBI mice. Increased hypertrophic **d** and bushy **e** and decreased ramified **c** Iba1+ microglia were observed in the ipsilateral hippocampus of Sham+DSS mice compared to Sham mice. Data expressed as mean ± s.e.m (*cohort* 2, *n* = 7–10/group). **b, c** * *p* < 0.5 vs Sham, ** *p* < 0.01 vs Naïve, **** *p* < 0.0001 vs Naïve/Sham, ## *p* < 0.01 vs Sham+DSS, #### *p* < 0.0001 vs Sham+DSS, ^ *p* = <0.05 vs Naïve+DSS, ^^ *p* < 0.01 vs Naïve+DSS, ^^^^ *p* < 0.0001 vs Naïve+DSS, + *p* < vs Sham or TBI, ++ *p* < 0.01 vs Sham

To assess the effect of intestinal inflammation on neuroinflammation, we performed unbiased stereological assessment of Iba1+ cells to quantify microglial cell number and activation state in the ipsilateral hippocampus at 1 week DSS recovery, as previously described [2, 43]. Microglial phenotype is closely related to their functional state with surveilling microglia having ramified morphology and highly reactive microglia exhibiting a bushy or hypertrophic morphology [43]. TBI increased the total number of microglia present in the ipsilateral hippocampus compared to Naïve and Sham mice (TBI effect: F_(2, 45)_ = 37.62, *p* < 0.0001; 16316.5 ± 779.6 cells/mm^3^ TBI vs 10864.1 ± 366.2 cells/mm^3^ Naïve, *p* = 0.0012 and 11697.6 ± 828.6 cells/mm^3^ Sham, *p* = 0.0111; Fig. 9b). Total number of microglia were not significantly increased in TBI + DSS mice compared to TBI mice (DSS effect: F_(1, 46)_ = 2.056, *p* = 0.1584; 21313.1 ± 2410.4 cells/mm^3^, *p* = 0.1537 vs TBI; Fig. 9b). Morphological analysis revealed that TBI increased the number of activated microglia (mean ± s.e.m.; hypertrophic microglia: TBI effect: F_(2, 45)_ = 63.78, *p* < 0.0001; 5389.4 ± 513.8 cells/mm^3^ TBI vs 1006.6 ± 230.5 cells/mm^3^ Naïve, *p* < 0.0001 and 1299.5 ± 201.3 cells/mm^3^ Sham, *p* < 0.0001; bushy microglia: TBI effect: F_(2, 45)_ = 76.42, *p* < 0.0001; 2027.9 ± 207.3 cells/mm^3^ TBI vs 203.2 ± 48.0 cells/mm^3^ Naïve, *p* < 0.0001 and 350.1 ± 65.7 cells/mm^3^ Sham, *p* < 0.0001; Fig. 9d, e), while the number of ramified microglia remained unchanged compared to Naïve and Sham mice (TBI effect: F_(2, 45)_ = 4.299, *p* = 0.0194; 8899.2 ± 338.3 cells/mm^3^ TBI vs 9654.3 ± 416.0 cells/mm^3^ Naïve, *p* = 0.9955 and 10048.0 ± 782.2 cells/mm^3^ Sham, *p* = 0.6985; Fig. 9c). The number of activated microglia was further shifted with DSS administration in TBI mice. TBI + DSS mice had similar levels of hypertrophic microglia (DSS effect: F _(1, 45)_ = 15.55 *p* = 0.0003; 5658.8 ± 1650.8 cells/mm^3^, *p* < 0.3594 vs TBI; Fig. 9d) but significantly increased bushy microglia (5686.8 ± 1650.8 cells/mm^3^, *p* < 0.0287 vs TBI, Fig. 9e), with a decrease in ramified microglia compared to TBI mice (DSS effect: F_(1, 46)_ = 16.94 *p* = 0.0002, 6618.1 ± 619.2 cells/mm^3^, *p* < 0.0435 vs TBI; Fig. 9c). Notably, DSS treatment in Sham-injured mice (Sham+DSS) increased the number of activated microglia compared to Sham mice (3434.7 ± 491.6 hypertrophic cells/mm^3^_,_ *p* < 0.0031 vs Sham; 981.5 ± 104.1 bushy cells/mm^3^_,_ *p* < 0.0075 vs Sham). This increase in activated microglia in Sham+DSS mice was associated with a decrease in ramified microglia compared to Sham mice (7580.4 ± 371.1 ramified cells/mm^3^, *p* < 0.0283 vs Sham; Fig. 9c), while total number of microglia present remained comparable to Sham levels (Fig. 9b). No changes were found between Naïve and Naïve+DSS mice (8946.6 ± 572.7 ramified cells/mm^3^, *p* < 0.9400 vs Naïve; 1262.9 ± 290.3 hypertrophic cells/mm^3^, *p* = 0.9463 vs Naive; 296.6 ± 86.8 bushy cells/mm^3^, *p* < 0.4648 vs Naive; Fig. 9b-e**)**. Increased microglial activation was not observed in the contralateral hippocampus in DSS-treated mice (data not shown). This analysis of microglial activation suggests that intestinal inflammation during chronic TBI induces, or exacerbates, hippocampal neuroinflammation in Sham-injured and TBI mice, respectively, which may contribute to the observed neurodegeneration and neurobehavioral deficits in these mice.

### Intestinal inflammation alters hippocampal expression of genes related to neuropathology, neuroinflammation, neuronal structure, and neurotransmission in Sham-injured mice

**Fig. 10:**
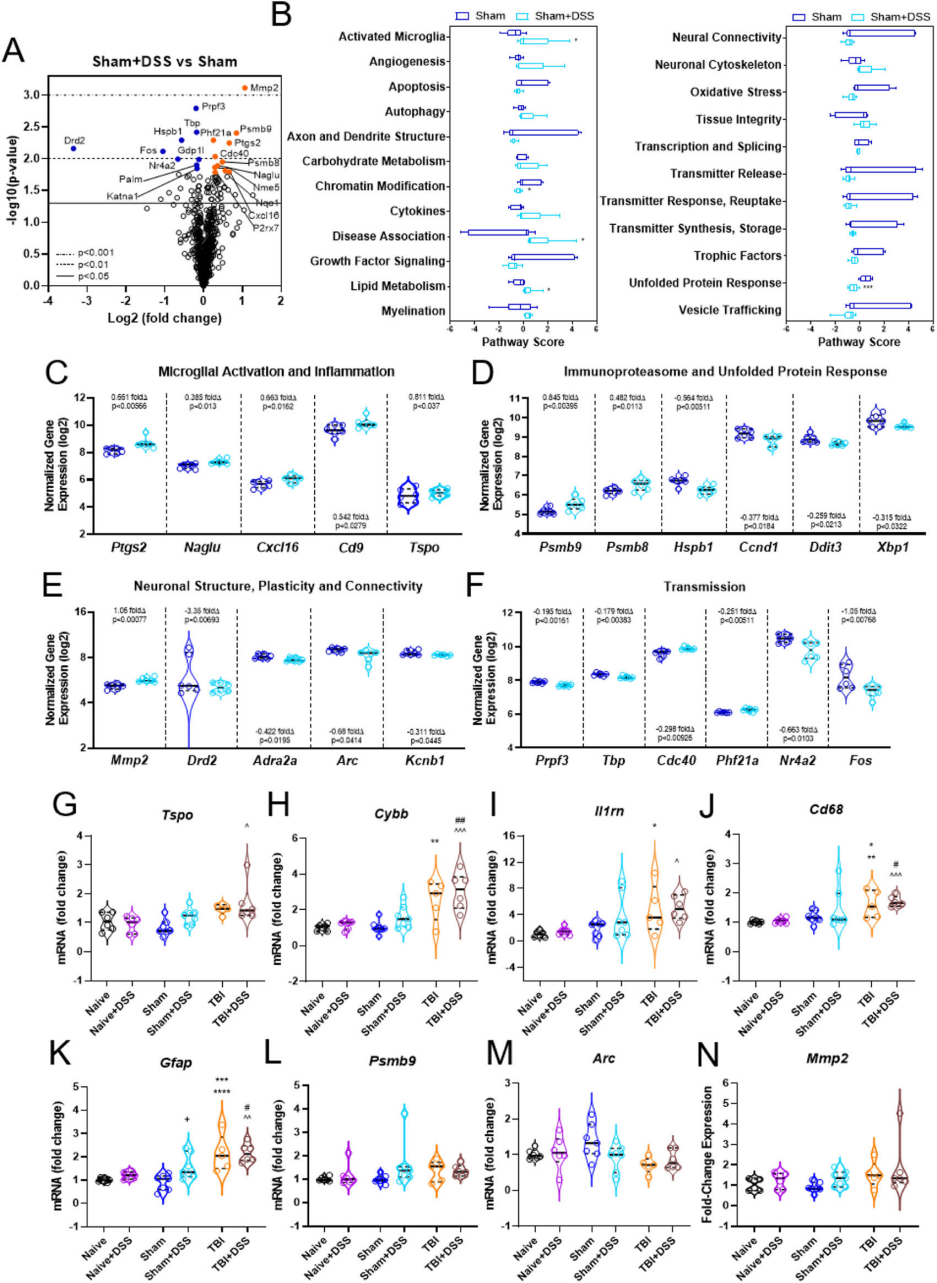
Intestinal inflammation alters hippocampal expression of neuropathology, neuroinflammation, and neurotransmission-related genes in Sham-injured mice. Volcano plot of differential gene expression between Sham and Sham+DSS mice **a**. Pathway expression scores comparing Sham and Sham+DSS mice. Scores for activated microglia, disease association and lipid metabolism were significantly increased in Sham+DSS mice **b**, (left panel), while unfolded protein response and chromatin modification was decreased in Sham+DSS mice **b**, (right panel). Normalized gene expression changes in Sham+DSS vs Sham mice for genes involved in microglial activation and inflammation **c**, the immunoproteasome and unfolded protein response **d**, neuronal structure, plasticity, and connectivity **e** and neurotransmission **f**. qPCR confirmation of selected genes associated with neuroinflammation, (*Tspo*, *Cybb*, *Il1rn*, *Psmb9*, *Cd68*, *Gfap*; **g–k**) and neuronal structure, plasticity, and connectivity (*Mmp 2* and *Arc*; **m**, **n**) in all experimental groups. Pathway scores, normalized gene expression and qPCR data expressed as mean ± s.e.m (*n* = 5–7/group). **b** * *p* < 0.05 vs Sham, *** *p* < 0.001 vs Sham. **g–n** * *p* < 0.05 vs Naïve/Sham, ** *p* < 0.01 vs Naïve/Sham, *** *p* < 0.001 vs Naïve, **** *p* < 0.0001 vs Sham, ^ *p* < 0.05 vs Naïve+DSS, ^^ *p* < 0.01 vs Naïve+DSS, ^^^ *p* < 0.001 vs Naïve+DSS, # *p* < 0.05 vs Sham+DSS, ## *p* < 0.01 vs Sham+DSS, + *p* < 0.005 vs Sham. Abbreviations: ; *Adra2a*, Adrenoceptor Alpha 2A; *Arc*, activity-regulated cytoskeleton-associated protein; *Tspo*, translocator protein; *Ccnd1*, cyclin D1*; Cd68*, cluster of differentiation 68; *Cd9*, cluster of differentiation 9; *Cdc40*, cyclin division cycle 40; *Cxcl16*, chemokine ligand 16; *Cybb*, cytochrome b-245 beta chain; *Ddi3t*, DNA Damage Inducible Transcript 3; *Drd2*, dopamine receptor D2; *Fos*, fos proto-oncogene; *Gfap*, glial factor activating protein; *Hsbp1*, heat-shock binding protein 1; *Il1rn*, interleukin-1 receptor antagonist; *Kcnb1*, potassium voltage-gated channel subfamily B member 1; *Mmp2*, matrix metalloprotease 2; *Naglu*, alpha-N-acetylglucosaminidase; *Nr4a2*, nuclear receptor subfamily 4 group A member 2; *Phf21a*, PHD finger protein 21A; *Psmb8*,*9*, proteasome 20S subunit beta 8,9; *Ptgs2*, prostaglandin-endoperoxide synthase 2; *Prpf3*, pre-mRNA processing factor 3; *Tbp*, TATA box binding protein; *Xbp1*, X-box binding protein 1

As the largest behavioral and neuropathological differences occurred in Sham vs Sham+DSS mice, we next assessed gene expression changes in these mice at 4 weeks post DSS administration. Differential gene expression changes in the ipsilateral hippocampus in Sham and Sham+DSS mice were assessed using NanoStringDiff, with significance defined as a *p* value < 0.05. The top twelve genes satisfying this criterion included *Drd2*, *Fos*, *Nr4a2*, *Hspb1*, *Gdp1l*, *Tbp*, *Prpf3*, *Phf21a*, *Cdc40*, *Ptgs2*, *Psmb9*, and *Mmp2*, with *Mmp2* and *Drd2* exhibiting log2 fold changes of over 1 or less than − 1 (Fig. 10a). The remaining genes showing significant changes in expression were subtler in overall log2 fold change. Next, a pathway analysis was run by NanoStringDiff, grouping gene changes between Sham and Sham+DSS mice into pathways based on known biological functions. Sham+DSS mice exhibited significantly increased pathway scores for activated microglia (*t*_(10)_ = 2.016, *p* = 0.0357 vs Sham), chromatin remodeling (*t*_(10)_ = 1.875, *p* = 0.0452 vs Sham), disease association (*t*_(10)_ = 2.041, *p* = 0.0342 vs Sham), and lipid metabolism (*t*_(10)_ = 2.266, *p* = 0.0235 vs Sham) and a significant decrease in the pathway score for unfolded protein response (*t*_(10)_ = 4.227, *p* = 0.0009 vs Sham) (Fig. 10b). Specific analysis of normalized gene expression for selected genes involved in neuroinflammation and neuropathology demonstrated alterations in key genes involved in microglia activation (Fig. 10c), the immunoproteasome (Fig. 10d), neuronal structure, plasticity, and connectivity (Fig. 10e), and neuronal transmission (Fig. 10f) by DSS administration.

Select genes identified by either nanostring or known to be involved in neuroinflammation (*Tspo*, *Cybb*, *Il1rn*, *Cd68*, *Gfap*, *Psmb9*) and neuropathology (*Mmp2*, *Arc*) were confirmed further in all experimental groups by qPCR using mRNA isolated from the ipsilateral hippocampus in all experimental groups. TBI alone increased expression of *Cybb*, *Il1rn*, *Cd68*, and *Gfap* compared to Naïve mice (TBI effect; *Cybb:* F_(2, 31)_ = 21.69, *p* < 0.0001; *Il1rn:* F_(2, 31)_ = 9.071, *p* < 0.0001; *Cd68:* F_(2, 31)_ = 15.42, *p* < 0.0001; *Gfap:* F_(2, 31)_ = 20.18, *p* < 0.0001; Fig. 10g-k). *Tspo*, *Psmb9*, and *Mmp2* expression were not significantly upregulated in TBI mice (TBI effect; *Tspo:* F_(2, 31)_ = 7.446; *Psmb9:* F_(2, 31)_ = 23.07, *p* = 0.1164; *Mmp2:* F_(2, 31)_ = 3.255, *p* = 0.0521; Fig. 10l, m). DSS did not further significantly upregulate expression of *Tspo*, *Cybb*, *Il1rn*, *Cd68*, *Gfap*, *Pmsb9*, or *Mmp2* in TBI + DSS compared to TBI mice at this time point (DSS effect; *Tspo:* F_(2, 31)_ = 1.940, *p* = 0.1736; *Cybb:* F_(2, 31)_ = 3.3669, *p* = 0.0762; *Il1rn:* F_(2, 31)_ = 2.373, *p* < 0.1336; *Cd68:* F_(2, 31)_ = 1.992, *p* < 0.1685; *Gfap:* F_(2, 31)_ = 4.389, *p* < 0.0444; *Psmb9:* F_(2, 31)_ = 3.753, *p* = 0.0619; *Mmp2:* F_(2, 31)_ = 2.654, *p* = 0.1134; Fig. 10g-n). The expression levels of *Tspo*, *Cybb*, *Il1rn*, *Cd68*, *Gfap*, *Pmsb9*, and *Mmp2* were increased in Sham+DSS mice compared to Sham; however, these increases were not significant (Fig. 10g-n). *Arc* appeared as a hit on the nanostring panel, yet qPCR confirmation showed no significant differences between the experimental groups (TBI × DSS effect: F_(2, 31)_ = 2.076, *p* = 0.1425; Fig. 10m). The network gene analyses of ipsilateral hippocampus shows that acute colitis in Sham mice affected a variety of transcriptional pathways involved in neuroinflammation and neuropathology. Confirmation qPCR in the ipsilateral hippocampus demonstrated that acute colitis following craniotomy (Sham+DSS) resulted in a trend for increased expression of neuroinflammatory (*Tspo*, *Cybb*, *Gfap*, *Psmb9*) and neuropathology (*Mmp2*) genes compared to water-treated Sham mice. TBI increased microglia-related inflammatory (*Cybb*, *Cd68*, *Psmb9*) and anti-inflammatory (*Il1rn*) genes, as well as genes related to astrocyte reactivity (*Gfap*) even 4 weeks following DSS administration consistent with the observed pathology in the ipsilateral hippocampus in these mice.

### Intestinal inflammation results in a sustained extraintestinal and systemic immune response

**Fig. 11:**
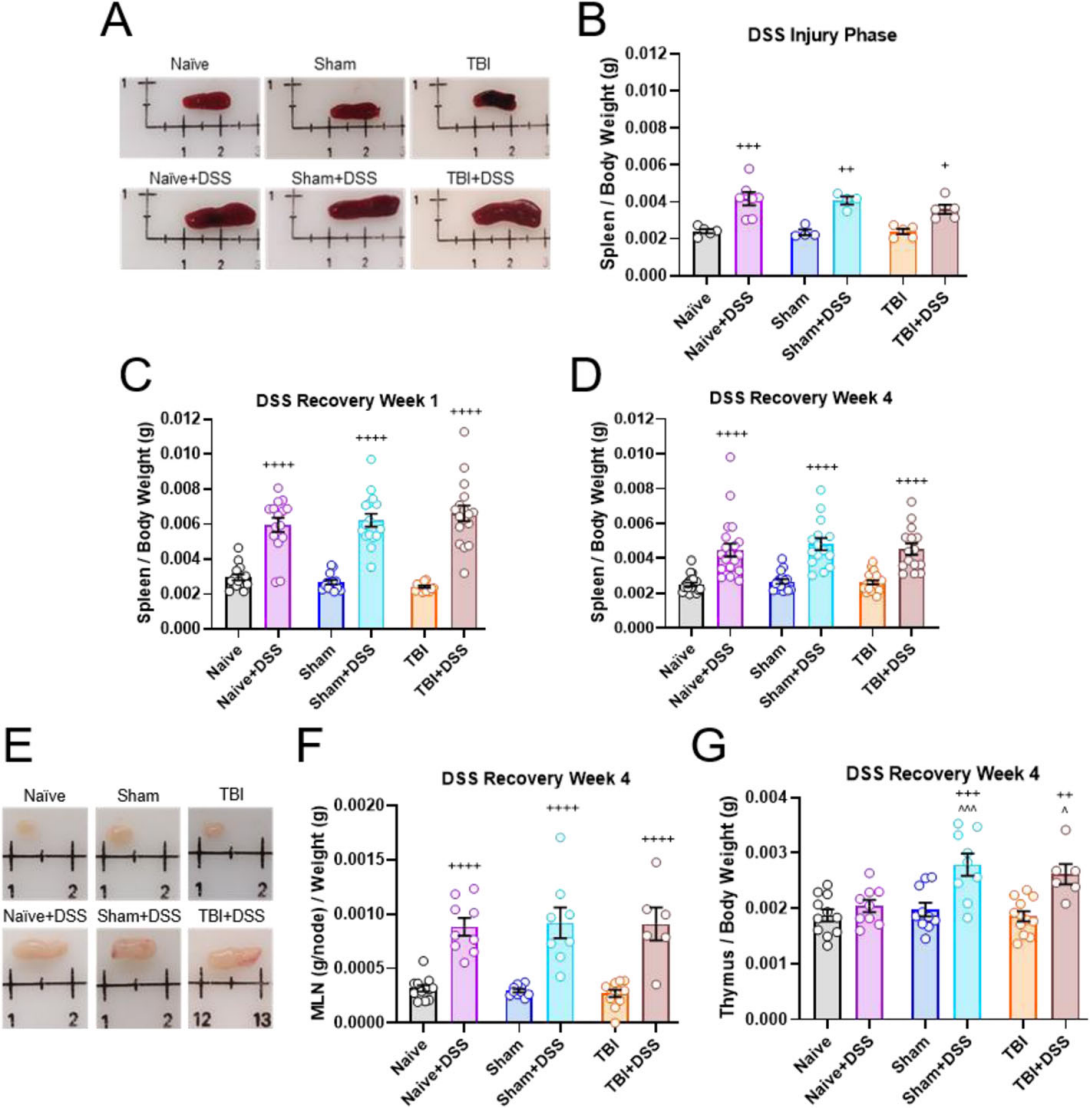
DSS administration results in a sustained extraintestinal and systemic immune response. Representative images of spleens collected at the end of the fourth week of the DSS recovery phase (*cohort* 3). , **a **Acute DSS administration resulted in a significant increase in spleen weights in all mice beginning during the DSS injury phase (*cohort* 1), **b** that persisted through the end of the first (*cohort* 2, **c**) and fourth weeks (*cohort* 3), **a**, **d** of the DSS recovery phase. Representative images of mesenteric lymph nodes (mLNs) collected at the end of the fourth week of the DSS recovery phase (*cohort* 3). , **e**. A significant increase in mLN weight was found at the end of the fourth recovery week in all DSS groups (*cohort* 3), **f**. Sham+DSS and TBI + DSS mice also exhibited increased thymus weight at the end of the fourth DSS recovery week compared to Sham and TBI mice, respectively (*cohort* 3), **g**. Data expressed as mean ± s.e.m (*n* = 4–7/group *cohort* 1 spleens; *n* = 14–17/group *cohort* 2 spleens; *n* = 15–21/group *cohort* 3 spleens; *n* = 6–12/group mLNs, thymus). **a–f** + *p* < 0.05 vs TBI, ++ *p* < 0.01 vs Sham, +++ *p* < 0.001 vs Naïve, ++++ *p* < 0.0001 vs Naïve, Sham, or TBI. **g** ^ *p* < 0.05 vs Naïve+DSS, ^^^ vs Naïve+DSS; ++ *p* < 0.01 vs TBI, +++ *p* < 0.001 vs Sham

Acute administration of DSS induces a local (intestinal), extraintestinal (mLNs), and peripheral (spleen, blood) immune responses in rodents [57]. To assess if a systemic immune response was still present during the later stages of DSS recovery, we weighed the thymus, spleens, and mLNs at the end of the fourth DSS recovery week. All organ weights were normalized to individual mouse body weight. Spleen to body weight ratios increased for all DSS-treated mice compared to their water-treated counterparts beginning during the DSS injury phase (DSS effect: F_(1, 24)_ = 52.16, *p* < 0.0001, Fig. 11b). This increase in spleen/body weight continued into the first week of the DSS recovery phase (DSS effect: F_(1, 85)_ = 295.1, *p* < 0.0001; Fig. 11c) and through the fourth week of the DSS recovery phase (DSS effect: F_(1, 103)_ = 111.9, *p* < 0.0001; Fig. 11d). Corresponding with the increase in spleen/body weights, DSS also increased mLN size and weight beginning during the DSS injury phase and persisting through the end of the first week of the recovery phase (data not shown). This increase in mLN size and weight was sustained through the end of the fourth DSS recovery week (DSS effect: F_(1, 51)_ = 107.8, *p* < 0.0001; Fig. 11e, f). Thymus size and weights were also increased in Sham+DSS and TBI + DSS mice compared to Sham and TBI mice at the end of the fourth DSS recovery week (DSS effect: F_(1, 51)_ = 27.08, *p* < 0.0001; Fig. 11g).

We next performed flow cytometry on colon, mLNs, spleen, and blood collected at the end of the fourth week of the DSS recovery phase for a more detailed examination of immune composition. Myeloid (CD11b+) cells were increased in the blood, spleen, mLN, and colon in DSS-treated mice compared to water-treated counterparts (DSS effect: Blood: F_(1, 25)_ = 15.92, *p* = 0.0005; Spleen: F_(1, 24)_ = 15.92, *p* = 0.0005; mLN: F_(1, 21)_ = 5.300, *p* = 0.0317; Colon: F_(1, 25)_ = 10.35, *p* = 0.0036). Circulating monocytes (CD45+ CD11b+ Ly6C^hi^+ CD115+) were comparably elevated between all DSS mice (DSS effect: F_(1, 25)_ = 10.93, *p* = 0.0029; Table 2). Circulating neutrophils (CD45+ CD11b+ Ly6C- Ly6G+) were also similarly increased in all DSS groups (DSS effect: Blood: F_(1, 25)_ = 15.95, *p* = 0.0005; Table 2). In the spleen, monocyte and neutrophil levels were higher in all DSS mice (DSS effect: Monocytes: F_(1, 24)_ = 11.74, *p* = 0.0022; Neutrophils: F_(1, 24)_ = 15.95, *p* = 0.0006), although this increase was most pronounced in the Naïve+DSS mice relative to the Sham+DSS and TBI + DSS mice (Table 2). Lymphocytes (CD45+ CD11b- CD3+) were not altered in any of the experimental groups in the blood or the spleen (DSS effect: Blood: F_(1, 25)_ = 1.981, *p* = 0.1716; Spleen: F_(1, 23)_ = 0.192, *p* = 0.6658; Table 2). In contrast, an increase in lymphocytes was observed in the mLN in mice administered DSS (DSS effect: F_(1, 21)_ = 5.118, *p* = 0.0344). B cell levels (CD45+ CD11b- MHCII+) were elevated in the mLN of all DSS mice (DSS effect: F_(1, 21)_ = 5.118, *p* = 0.0344) with no significant differences among the groups (Table 2). CD4+ and CD8+ T lymphocytes were also increased in the colon in DSS mice compared to water-treated mice, with no significant differences among the DSS groups (DSS effect, CD4+: F_(1, 25)_ = 1.399, *p* = 0.248; CD8+: F_(1, 25)_ = 0.911, *p* = 0.342; Table 2). Combined, these results demonstrate that DSS administration results in a prolonged systemic immune response across all groups, which may contribute to observed neurobehavioral deficits, neurodegeneration, and neuroinflammation and illustrate the susceptibility of Sham and TBI mice to enteric challenge.

**Table 2:**
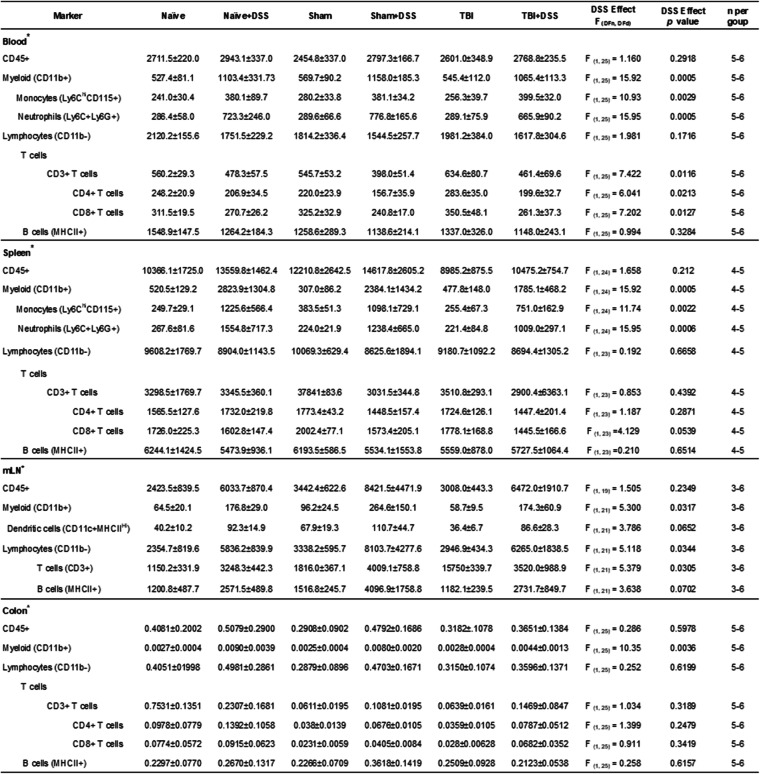
Immune characterization of the blood, spleen, mesenteric lymph nodes, and colon. Flow cytometry was performed on tissue collected at the end of the fourth DSS recovery week (PTD64-67). Data expressed as mean ± s.e.m. (*n* = 3–6/group). Cells/μl in blood (*), cells/μl/mg tissue (^), cells/μl/number of mLN (+)

### Dysautonomia is transiently induced acutely after TBI and is later persistently reactivated in Sham-injured and TBI mice subjected to intestinal inflammation

**Fig. 12:**
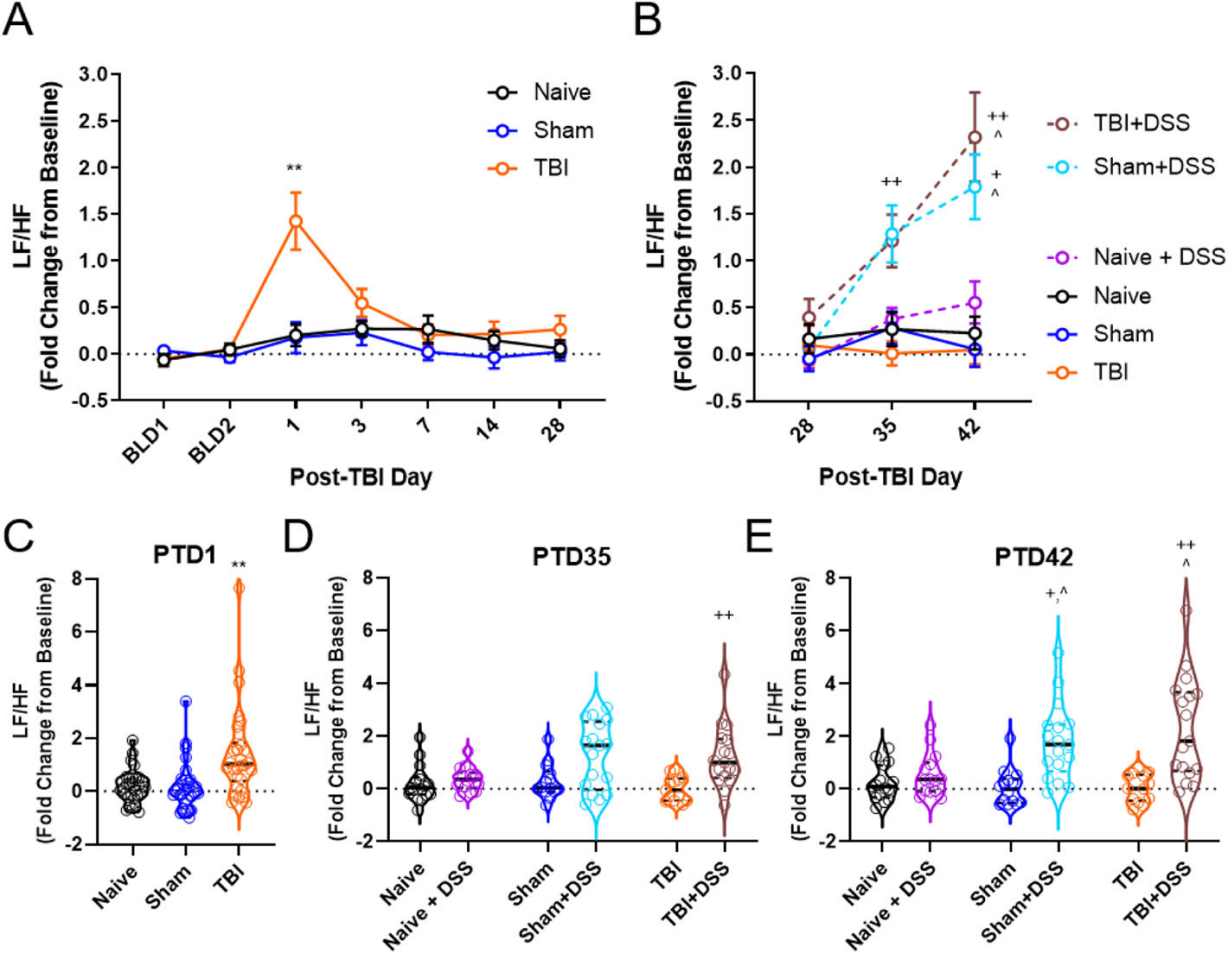
Dysautonomia is induced acutely by TBI and persistently by intestinal inflammation during chronic TBI. TBI significantly and transiently elevated sympathetic tone (↑LF/HF) at 24 h returning to baseline levels by 7 days post-TBI injury **a**. Sham+DSS and TBI + DSS mice exhibited increased sympathetic tone during both DSS injury and 1-week recovery phases compared to their water-treated counterparts (Sham, TBI) and Naïve+DSS mice **b**. Graphs of LF/HF ratios at specific time points within the study; PTD1 **c**, PTD35 **d**, and PTD42 **e**. Data expressed as mean ± s.e.m (pre-DSS: *n* = 30–31/group; DSS injury phase: *n* = 14–17/group DSS injury; 1-week recovery phase: *n* = 14–17/group). **a–e** ** *p* < 0.01 vs Naïve/Sham, ^ *p* < 0.05 vs Naïve+DSS, + *p* < 0.05 vs Sham, ++ *p* < 0.01 vs TBI. Abbreviations: LF, low frequency; HF, high frequency

Inflammatory bowel disease and TBI are known to alter autonomic balance in patients [58–61]. We performed electrocardiography to measure heart rate variability (HRV) and assess changes in autonomic balance due to TBI and intestinal inflammation during chronic TBI. Assessment of HRV time- and frequency-domains by power spectral analyses allows for the evaluation of sympathetic and parasympathetic activity [62]. In this study, power spectral analysis of the frequency domain was performed to calculate HRV low-frequency (LF) and high-frequency (HF) values. LF values represent the sympathetic input, whereas HF values represent both sympathetic and parasympathetic (vagal) input. Increases in LF/HF ratios suggest an increase in sympathetic tone, while decreases in LF/HF suggest an increase in parasympathetic tone. As expected, TBI resulted in an acute, transient, increase in LF/HF ratios compared to Sham and Naïve mice, indicating a shift to sympathetic dominance. Within 24 h of brain injury, LF/HF ratios were significantly elevated compared to Naïve and Sham mice (mean ± s.e.m.; F_(12,514)_ = 4.981, *p* < 0.0001; TBI:1.42 ± 0.31 LF/HF, Naïve:0.20 ± 0.12 LF/HF, Sham:0.18 ± 0.17 LF/HF, *p* = 0.0019 vs Naïve, *p* = 0.0025 vs Sham; Fig. 12a, c). This increase in sympathetic tone (↑LF/HF) returned to baseline levels within 7 days of brain injury. At 28 days post brain injury, no significant differences in LF/HF ratios were observed between Naïve, Sham, and TBI mice (TBI:0.27 ± 0.15 LF/HF, Naïve:0.06 ± 0.09 LF/HF, Sham:0.02 ± 0.09 LF/HF, *p* = 0.4513 vs Naïve, *p* = 0.3464 vs Sham; Fig. 12a, d). However, upon DSS administration, LF/HF ratios increased in TBI + DSS mice compared to TBI mice. By the end of the DSS injury phase, TBI + DSS mice had significantly elevated LF/HF ratios (F_(10,168)_ = 2.233, *p* < 0.0001; 0.01 ± 0.13 TBI vs 1.21 ± 0.28 TBI + DSS, *p* = 0.0097; Fig. 12b, d). Increased LF/HF ratios persisted through the end of the first week of the DSS recovery phase (0.05 ± 0.19 TBI vs 2.32 ± 0.47 TBI + DSS, *p* = 0.0024; Fig. 12b, e). Remarkably, LF/HF ratios were also increased in Sham+DSS mice at the end of the DSS injury phase compared to Sham mice (0.28 ± 0.17 Sham vs 1.28 ± 0.31 Sham+DSS, *p* = 0.0835; Fig. 12b, d). Sham+DSS showed a significant increase in sympathetic tone at the end of the first DSS recovery week compared to Sham mice (0.05 ± 0.19 Sham vs 1.79 ± 0.35 Sham+DSS, *p* = 0.0026; Fig. 12b, e). In contrast, Naïve+DSS mice exhibited no change in LF/HF ratios in response to DSS when compared to Naïve mice indicating that DSS alone does not induce dysautonomia (0.23 ± 0.17 Naïve vs 0.55 ± 0.22 Naïve+DSS, *p* = 0.8531; Fig. 12b, e). The pro-inflammatory effects of a dominant sympathetic tone induced by intestinal inflammation following Sham craniotomy or TBI may contribute to observed neurobehavioral, neuropathological, and systemic immune response changes.

## Discussion

TBI is a chronic disease process [63, 64] that can be influenced by subsequent systemic challenges [65]. We previously showed that enteric infection during chronic TBI worsened posttraumatic lesion volume and neuroinflammation [24], but the mechanisms of this brain-gut effect and long-term effects on neurological function are unknown. This study is the first to report that induction of acute chemical colitis in male mice during chronic TBI exacerbates neurodegeneration and induces sustained impairments in fine motor coordination, social behavior, and increases anxiety-like behavior. Importantly, similar colitis-induced posttraumatic sequelae were observed in animals subjected to craniotomy alone. Although commonly used as a sham injury control in TBI studies, craniotomy causes an acute, mild brain injury [25–28]. DSS administration following craniotomy induced sustained deficits in fine motor coordination, declarative memory, spatial learning and memory, social behavior and anxiety-like behavior, induced neurodegeneration, and increased microglia activation in the ipsilateral hippocampus. These DSS-induced neurobehavioral and neuropathological changes in Sham mice (Sham+DSS) were similar in magnitude and severity to those observed after TBI alone. Therefore, our findings provide further evidence that craniotomy is a mild injury and, as such, suggest that even mild TBI can enhance the susceptibility of the brain to a subsequent pro-inflammatory enteric challenge.

Preclinical and clinical studies indicate that TBI causes structural and functional damage to the GI tract [66, 67]. In rodents, TBI results in mucosal injury and impaired barrier function in the small intestine up to 72 h after injury [68–70] and 28 days after injury in the colon [24]. Enteric pathogen infection during chronic TBI did not alter the severity of infection or ability to clear the infection in mice [24]. Similarly, in the present study, DSS-induced changes in body weight and colonic damage were similar across all injury groups. Additionally, all DSS-treated mice recovered to comparable levels upon removal from DSS. These findings indicate that the deleterious effects of TBI on the gut do not affect its ability to withstand and recover from a pathogenic microbial infection [24] or deleterious chemical exposure.

Posttraumatic neuroinflammation and progressive neurodegeneration can last for years following the initial insult in TBI patients, contributing to sustained motor and cognitive deficits and increased risk of dementia [71–75]. Experimental TBI causes persistent impairments in fine motor coordination, declarative memory, and spatial learning and memory [76, 77]. Deficits in social recognition and memory were reported in pediatric and young adult mice subjected to TBI [45, 56, 78]. Inflammatory bowel disease (IBD) is also associated with neurological dysfunction in patients [55, 79, 80] and is a risk factor for neurodegenerative diseases and neurodevelopmental disorders [81–84]. Rodent models of acute colitis report deficits in working, declarative, and social recognition and memory [19, 22, 85]. In addition, psychiatric disorders are a feature of preclinical and clinical TBI and IBD [21, 86–90]. This study demonstrates that acute colitis during chronic TBI worsens long-term outcomes by exacerbating deficits in fine motor coordination, social behavior, and anxiety-like behavior. However, no additional deficits were observed in declarative memory, reflecting either a ceiling effect of TBI or that the NOR task is not sufficiently sensitive to detect compound changes in declarative memory induced by TBI and DSS.

Although other groups have found that acute DSS administration causes cognitive deficits and increased anxiety-like behavior in rodents [19, 22, 85], we found no such changes in our Naïve+DSS mice. Differences in our findings may be due to study design including (1) mice arriving 4 weeks prior to the induction of the TBI in order to allow for acclimation of the mice to the animal facility, behavioral rooms, experimenter handling; (2) minimizing the effects of the microbiome by utilizing a bedding mix protocol prior to beginning the study; and (3) behavioral testing being carried out at night under red lights during the animals wake cycle so as to minimize stress induced by disruption of their natural circadian rhythms.

TBI disrupts cortical, hippocampal, and limbic neuronal circuitry, altering mood and interfering with learning and memory and motor function [91, 92]. DSS administration was reported to decrease hippocampal neuronal activation and alter hippocampal neurogenesis [89, 93]. Moreover, chronic colitis preceding ischemic stroke in mice enlarged infarct volume and peri-lesional neuronal cell loss at 7 days postinjury [94]. We previously showed that infectious colitis increased posttraumatic lesion volume without altering hippocampal neuronal cell loss [24]. In the present study, DSS-induced colitis exacerbated hippocampal neurodegeneration in the DG hippocampal subregion in TBI + DSS mice compared to TBI mice, without affecting cortical lesion volume. Further work is required to address the neuropathological differences observed between enteric models.

Experimental TBI also induces chronic microglial activation up to 1 year following injury, contributing to progressive hippocampal neurodegeneration [2]. Inhibiting microglial activation or depleting microglia following experimental TBI improves long-term neurological outcomes [2, 36, 37, 54]. Infectious colitis after TBI increased peri-lesional activation of microglia and reactive astrocytes [24]. Acute DSS-induced colitis in mice also promotes cortical and hippocampal microglial activation in mice [93, 95, 96]. Here, DSS treatment increased chronic hippocampal microglial activation in TBI mice, concurrent with the loss of hippocampal DG neurons, suggesting a role for microglia in the neuropathological changes and neurobehavioral deficits observed. That acute colitis following craniotomy or TBI-induced neuronal cell loss and microglial activation specifically in the hemisphere of injury (ipsilateral) and not the uninjured hemisphere (contralateral) suggests that head injuries establish conditions locally that prime the brain for a deleterious response to subsequent peripheral challenges.

As the largest behavioral and neuropathological changes induced by acute colitis were observed in Sham-injured mice, network gene analysis was performed to examine the molecular changes between these two experimental groups (Sham vs Sham+DSS). Network-level gene analysis of the ipsilateral hippocampus from Sham and Sham+DSS mice using Nanostring Neuropathology mRNA array demonstrated that acute colitis upregulated the expression of genes associated with neurodegenerative disease and neuroinflammation while downregulating the expression of genes associated with neuronal structure, plasticity, transmission and connectivity, and the unfolded protein response. Quantitative PCR of the ipsilateral hippocampus further demonstrated that TBI increased microglia-related inflammatory and astrocyte reactivity-related genes. Acute colitis following craniotomy (Sham+DSS) did not result in a significant upregulation of microglia-related inflammatory genes compared to Sham mice. Further studies are needed to determine the time course of the molecular changes in the brain in response to acute colitis. Additionally, while Nanostring and qPCR provide interesting data on molecular changes occurring in the brain, these analyses were performed at the tissue level, which does not permit analysis of differential changes in the individual cell populations within the brain. The effect of colonic inflammation during chronic TBI on individual cell types, and sub-cell types, is an important consideration that will be investigated further in future studies. Importantly, this study demonstrates that there are long-term effects of intestinal inflammation at the molecular level in the brain which correlate with observed changes in neurodegeneration and microglial-related neuroinflammation.

The cholinergic anti-inflammatory pathway, involving both the vagus nerve and the spleen, mediates systemic and intestinal immune responses by inhibiting macrophages and pro-inflammatory cytokine production. Vagal stimulation induces an anti-inflammatory response in the spleen, reducing activated immune cells and pro-inflammatory cytokine production, whereas interruption of this vagal-spleen axis promotes pro-inflammatory immune responses [97–101]. Vagal simulation can reduce enteric inflammation independent of the spleen by attenuating local immune responses [102, 103]. Dysautonomia, involving disruption of sympatho-vagal balance, is correlated with increased morbidity and mortality in TBI patients following moderate/severe injuries [61, 104]. Furthermore, chronic imbalance of sympatho-vagal pathways is implicated in IBD [58–60] and experimental models of colitis in rodents [105]. Acute colitis following craniotomy and TBI induced persistent dysautonomia in both Sham and TBI mice, but not in Naïve mice. The concomitant enlargement of the mesenteric lymph nodes and spleen in all groups is consistent with persistent local and systemic immune activation, respectively. These changes were associated with a sustained elevation of myeloid cells in blood, spleen, and colon, and myeloid and lymphoid cells in the mesenteric lymph nodes. Taken together, these data suggest that acute colitis during chronic TBI induces a long-term disruption in the anti-cholinergic pathway, providing a potential mechanism by which enteric inflammation can exert its effects on the injured brain.

Neuroinflammation has been linked to activation of systemic immune responses. In agreement with our findings of increased microglial activation induced by colonic inflammation following TBI, preclinical studies show that peripheral injections triggering a systemic immune response (e.g., lipopolysaccharide, interleukin 1 beta) at acute and chronic time points following experimental TBI induce microglial activation and exacerbated TBI-related neuropathology and neurological outcomes [106–110]. It has been proposed that intestinal inflammation promotes neuroinflammation though an activated systemic immune response. Pro-inflammatory factors released from circulating, activated immune cells in response to an enteric challenge could increase blood brain barrier permeability, allowing access of circulating immune cells and mediators to the brain that serve to activate microglia and trigger a neuroinflammatory response [111]. Additionally, deficits in the cholinergic system in the brain following TBI have been reported [112, 113], which may be further affected by the sustained systemic immune response. A full examination of the effects of colonic inflammation-induced systemic immune activation and altered autonomic balance on the individual cell types in the brain will be investigated in future studies.

This preclinical study highlights the chronic effects of an acute episode of chemical colitis on craniotomy (Sham) and TBI. Robust extraintestinal and systemic immune responses are initiated by a single episode of colitis that persists through the recovery period when macroscopic disease evaluation of the colon suggests full recovery. These sustained extraintestinal and peripheral immune responses are likely being driven by the subclinical immune response in the colon, and potentially contributing to the persistent microglial-related inflammation and neurodegeneration observed in the hippocampus of the injured brain hemisphere. Neurobehavioral deficits begin during the acute colitis injury phase and are sustained through the recovery phase, suggesting that behavior, and the neural pathways associated with these behaviors, are permanently altered during this acute colitis injury phase, or alternatively, being sustained by the ongoing neurodegeneration and neuroinflammation occurring simultaneously. Importantly, this study highlights the differences between how the uninjured brain responds to acute colitis and sustained systemic inflammation as opposed to how the injured brain (craniotomy, TBI) responds to this same challenge.

Our study has several limitations, particularly regarding biological sex and the microbiome, which are worthy of further consideration. The importance of sex as a biological variable in both TBI and intestinal inflammation has been highlighted in multiple preclinical and clinical studies [48, 114–120]. As female mice are less susceptible to the effects of DSS [30–32] and our previous study examined *Cr* infection during chronic TBI [24] in male mice, this study was limited to male mice only. Examination of potential sex differences in the response to an enteric challenge after TBI is an important question that will be examined in future studies utilizing an enteric challenge model that would better allow direct comparison of male and female animals. Alterations in the gut microbiome induced by TBI play a key role in chronic TBI disease progression [34, 121–123] and dysbiosis is also a causal factory in inflammatory GI disorders and diseases [124–126]. Although our experimental design attempted to minimize effects of the microbiome, we did not assess how microbiome changes due to TBI or DSS might affect outcomes. The microbiome as a contributing factor in intestinal inflammation-induced changes in systemic and CNS immunity and worsening of TBI-related neurological outcomes should be examined in future studies.

This preclinical study strongly supports the concept that a secondary pro-inflammatory systemic challenge during chronic TBI exacerbates long-term TBI outcomes, with potential mechanisms including both the neural and immune pathways of the brain-gut axis. Sustained systemic inflammation initiated by an enteric challenge likely enhances the risk of neurodegenerative and neuropsychiatric diseases. Our findings are consistent with literature regarding clinically relevant sickness behavior [127, 128], which occurs in response to systemic inflammation and includes changes in brain energy metabolism and neuroinflammation, affecting mood and cognition [129].

## Conclusions

In summary, this preclinical study demonstrates that induction of colitis 28 days after craniotomy or TBI results in sustained long-term neuropathological, neuroinflammatory, and neurobehavioral outcomes in male mice, with the neural and systemic pathways of the brain-gut axis serving as the mechanistic links. Recognition of such brain-gut interactions after head injury will lead to a better appreciation of systemic-brain interactions that can affect chronic care following TBI and facilitate development of novel therapeutic interventions.

## Supplementary Information

**Additional file 1: Supplemental Table 1**. Organ morphometric measurements at the end of the DSS injury, 1- and 4-week recovery phases. Prior TBI did not alter the severity of DSS injury in the colon or cecum. No significant differences were observed between DSS-treated mice in markers of DSS injury severity including colonic shortening, colon weight/body weight and cecum weight/body weight ratios. All DSS-treated mice exhibited similar recovery from DSS in terms of colonic re-lengthening, reductions of colon weight/body weight, and increases in cecum weight/body weight ratios. Data expressed as mean ± s.e.m. a = *p* = 0.0463 vs Naive, b = *p* = 0.0049 vs Sham, c = *p* = 0.0488 vs TBI, d = *p* = 0.0003 vs Naïve, e = *p* < 0.0001 vs Sham, f = *p* = 0.0171 vs TBI, g = *p* < 0.0001 vs Naïve, h = *p* < 0.0001 vs TBI, i = *p* = 0.0001 vs Sham, j = *p* = 0.0004 vs Naïve, k = *p* = 0.0027 vs TBI.

**Additional file 2: Supplemental Table 2**. Weight loss statistical significance values. Specific *p* values for weight loss comparisons in Fig. 2a between water-treated and DSS-treated mice.

**Additional file 3: Supplemental Figure 1**. Liquid intake of mice. Estimated liquid intake was monitored daily beginning on the first day of DSS administration through the end of the study. No significant differences were observed between the DSS administered mice in terms of average amount of DSS consumed during the injury phase or normal drinking water consumed during the recovery phase. Data expressed as mean ± s.e.m (*n* = 15-21/group). + *p* < 0.05, ++ *p* < 0.01, +++ *p* < 0.001, ++++ *p* < 0.0001 vs water-treated counterparts.

**Additional file 4: Supplemental Figure 2**. Preference for objects during the familiarization stage of novel object recognition testing. Object preference for familiarization stage conducted prior to DSS administration (A), during the DSS injury phase (B), and fourth week of the DSS recovery phase (C). Mice from all experimental groups explored both objects presented during the familiarization stage equally. Data represented as mean ± s.e.m (*n* = 31-42/group pre-DSS administration; *n* = 15-21/group DSS injury phase and fourth DSS recovery week).

**Additional file 5: Supplemental Figure 3**. Object exploration times during NOR testing were unaltered by TBI or intestinal inflammation. Total time spent exploring objects during the familiarization stage and novel object stage conducted prior to DSS administration (A, D), during the DSS injury phase (B, E) and in the fourth week of the DSS recovery phase (C, F). Mice from all experimental groups spent similar amounts of time exploring the objects presented during each stage. Data represented as mean ± s.e.m (*n* = 31-42/group pre-DSS administration; n = 15-21/group DSS injury phase and fourth DSS recovery week).

**Additional file 6: Supplemental Figure 4**. Stimulus mouse exploration times during SA testing were unaffected by TBI or intestinal inflammation. Total time spent the stimulus mouse/object or both stimulus mice during the sociability stage (A) and social novelty stage (B), respectively. Data represented as mean ± s.e.m (*cohort 3*, *n* = 13-21/group).

## Data Availability

The datasets used in this study are available from the corresponding author upon request.

## Abbreviations

ANS: Autonomic nervous system
BW: Beam walk
CA: Cornu ammonis
CCI: Controlled cortical impact
CNS: Central nervous system
*Cr*: *Citrobacter rodentium*
DAI: Disease activity index
DG: Dentate gyrus
DSS: Dextran sodium sulfate
ECG: Electrocardiography
GI: Gastrointestinal
HF: High frequency
HRV: Heart rate variability
IBD: Inflammatory bowel disease
LF: Low frequency
LDB: Light-dark box
MB: Marble burying
mLNs: Mesenteric lymph nodes
MWM: Morris water maze
NO: Novel object
NOR: Novel object recognition
PI: Preference Index
PTD: Post-TBI day
SA: Crawley’s three-chamber social approach task
TBI: Traumatic brain injury
VA: Visual acuity
