# Supplementary figures and images for: Acute colitis during chronic experimental traumatic brain injury in mice induces dysautonomia and persistent extraintestinal, systemic, and CNS inflammation with exacerbated neurological deficits
Source: J Neuroinflammation. 2021 Jan 18;18:24. doi: 10.1186/s12974-020-02067-x (PMC7814749; doi:10.1186/s12974-020-02067-x)

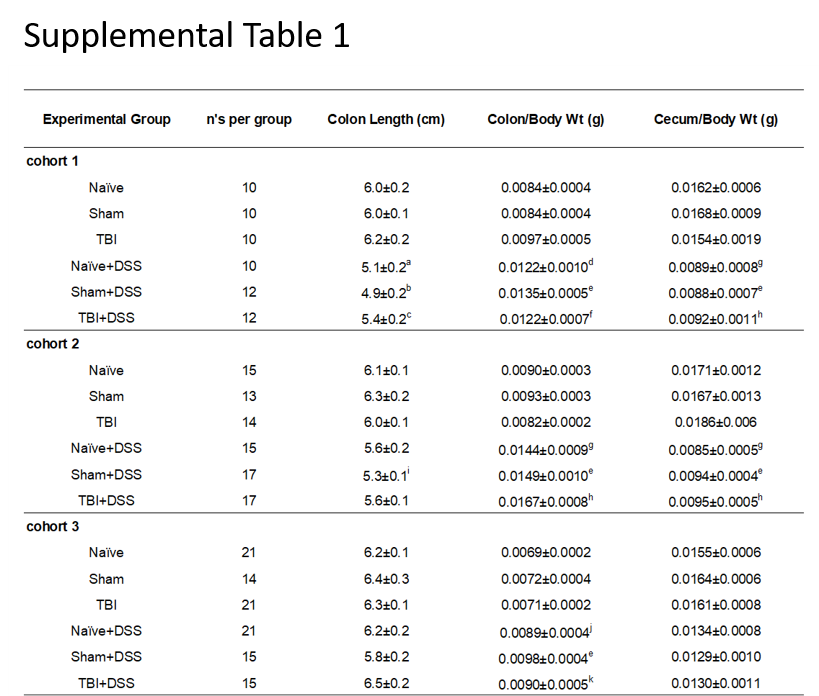

Supplement: Supplementary file 1 — Additional file 1: Supplemental Table 1. Organ morphometric measurements at the end of the DSS injury, 1- and 4-week recovery phases. Prior TBI did not alter the severity of DSS injury in the colon or cecum. No significant differences were observed between DSS-treated mice in markers of DSS injury severity including colonic shortening, colon weight/body weight and cecum weight/body weight ratios. All DSS-treated mice exhibited similar recovery from DSS in terms of colonic re-lengthening, reductions of colon weight/body weight, and increases in cecum weight/body weight ratios. Data expressed as mean ± s.e.m. a = p = 0.0463 vs Naive, b = p = 0.0049 vs Sham, c = p = 0.0488 vs TBI, d = p = 0.0003 vs Naïve, e = p < 0.0001 vs Sham, f = p = 0.0171 vs TBI, g = p < 0.0001 vs Naïve, h = p < 0.0001 vs TBI, i = p = 0.0001 vs Sham, j = p = 0.0004 vs Naïve, k = p = 0.0027 vs TBI. [file 12974_2020_2067_MOESM1_ESM.docx]

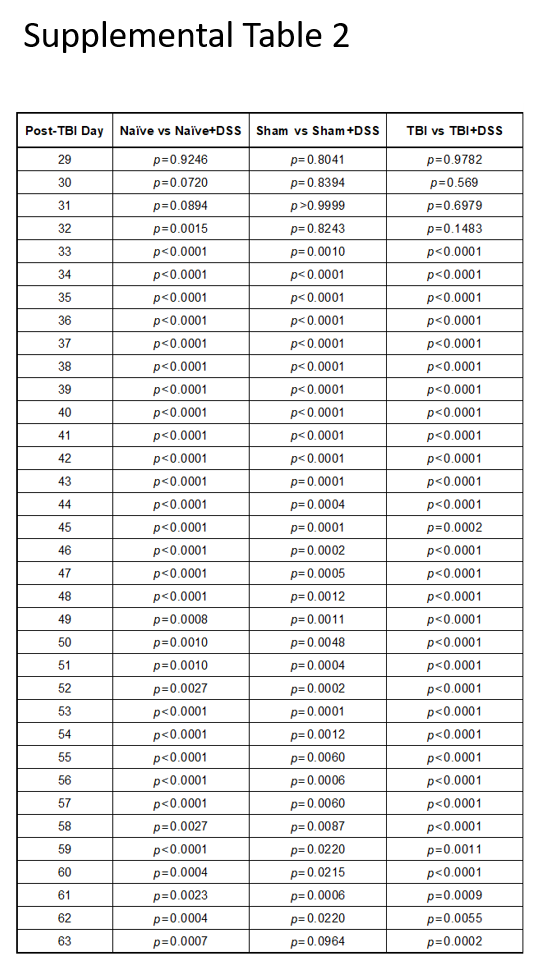

Supplement: Supplementary file 2 — Additional file 2: Supplemental Table 2. Weight loss statistical significance values. Specific p values for weight loss comparisons in Fig. 2a between water-treated and DSS-treated mice. [file 12974_2020_2067_MOESM2_ESM.docx]

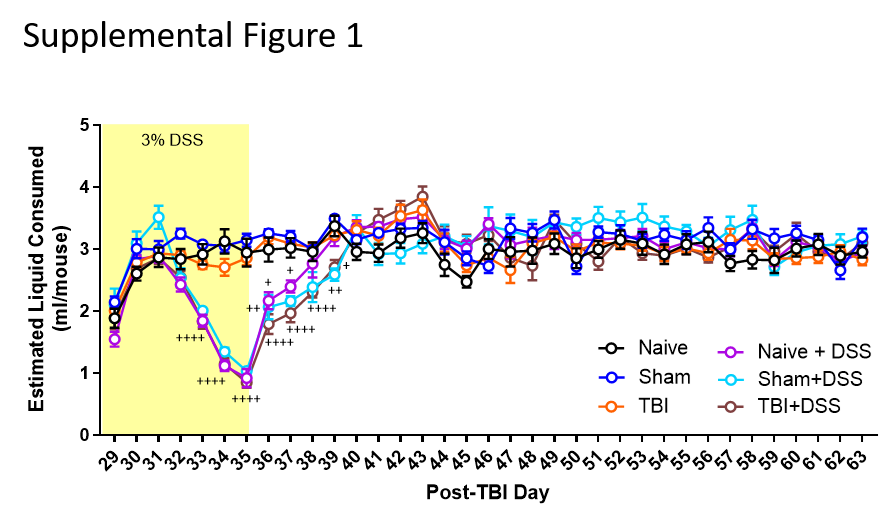

Supplement: Supplementary file 3 — Additional file 3: Supplemental Figure 1. Liquid intake of mice. Estimated liquid intake was monitored daily beginning on the first day of DSS administration through the end of the study. No significant differences were observed between the DSS administered mice in terms of average amount of DSS consumed during the injury phase or normal drinking water consumed during the recovery phase. Data expressed as mean ± s.e.m (n = 15-21/group). + p < 0.05, ++ p < 0.01, +++ p < 0.001, ++++ p < 0.0001 vs water-treated counterparts. [file 12974_2020_2067_MOESM3_ESM.docx]

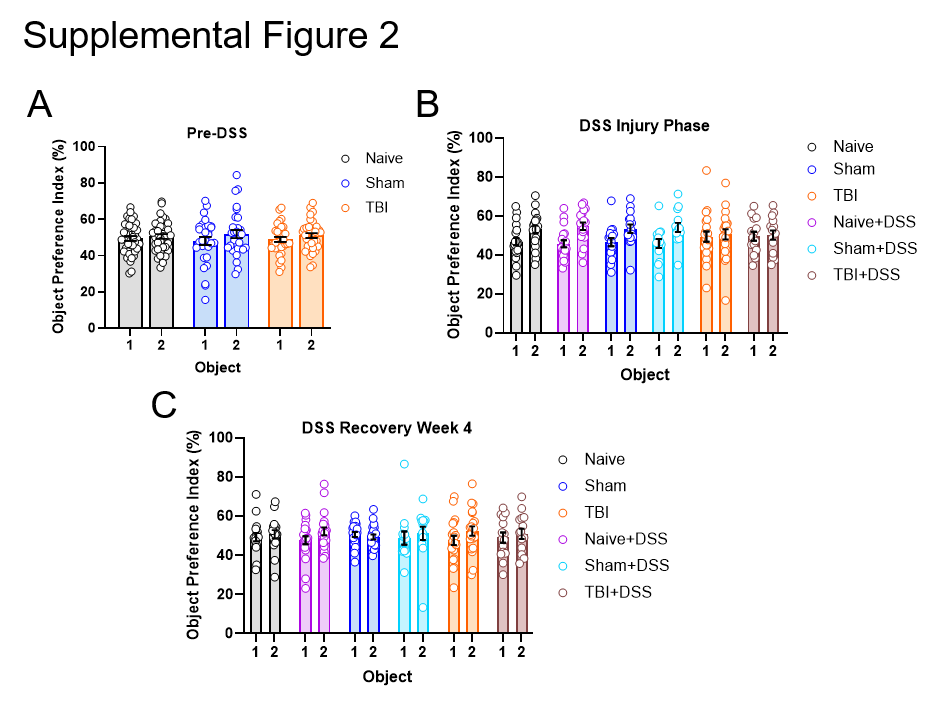

Supplement: Supplementary file 4 — Additional file 4: Supplemental Figure 2. Preference for objects during the familiarization stage of novel object recognition testing. Object preference for familiarization stage conducted prior to DSS administration (A), during the DSS injury phase (B), and fourth week of the DSS recovery phase (C). Mice from all experimental groups explored both objects presented during the familiarization stage equally. Data represented as mean ± s.e.m (n = 31-42/group pre-DSS administration; n = 15-21/group DSS injury phase and fourth DSS recovery week). [file 12974_2020_2067_MOESM4_ESM.docx]

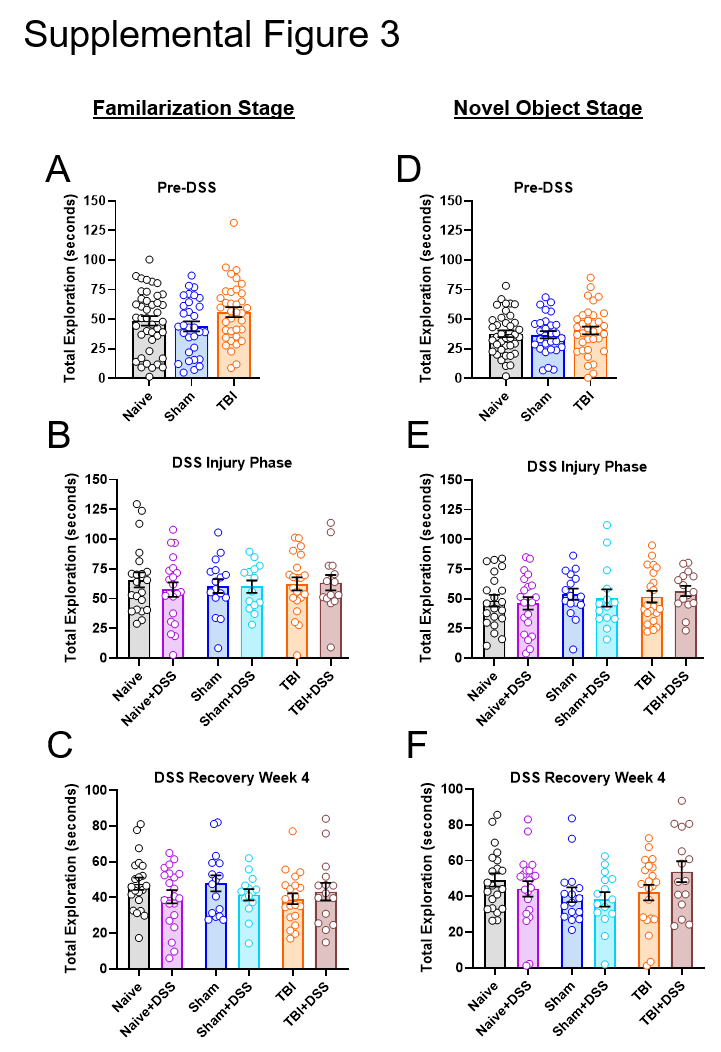

Supplement: Supplementary file 5 — Additional file 5: Supplemental Figure 3. Object exploration times during NOR testing were unaltered by TBI or intestinal inflammation. Total time spent exploring objects during the familiarization stage and novel object stage conducted prior to DSS administration (A, D), during the DSS injury phase (B, E) and in the fourth week of the DSS recovery phase (C, F). Mice from all experimental groups spent similar amounts of time exploring the objects presented during each stage. Data represented as mean ± s.e.m (n = 31-42/group pre-DSS administration; n = 15-21/group DSS injury phase and fourth DSS recovery week). [file 12974_2020_2067_MOESM5_ESM.docx]

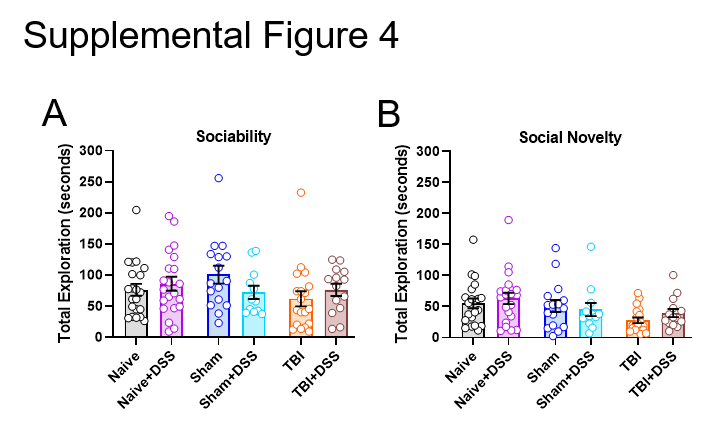

Supplement: Supplementary file 6 — Additional file 6: Supplemental Figure 4. Stimulus mouse exploration times during SA testing were unaffected by TBI or intestinal inflammation. Total time spent the stimulus mouse/object or both stimulus mice during the sociability stage (A) and social novelty stage (B), respectively. Data represented as mean ± s.e.m (cohort 3, n = 13-21/group). [file 12974_2020_2067_MOESM6_ESM.docx]
